# *In vivo* single-molecule imaging of RecB reveals efficient repair of DNA damage in *Escherichia coli*

**DOI:** 10.1093/nar/gkaf454

**Published:** 2025-06-04

**Authors:** Alessia Lepore, Daniel Thédié, Lorna McLaren, Louise Goossens, Benura Azeroglu, Oliver J Pambos, Achillefs N Kapanidis, Meriem El Karoui

**Affiliations:** Institute of Cell Biology, University of Edinburgh, EH9 3FF, Edinburgh, United Kingdom; Centre for Engineering Biology, University of Edinburgh, EH9 3BF, Edinburgh, United Kingdom; Laboratory for Optics and Biosciences, École Polytechnique, Institut Polytechnique de Paris, 91120, Palaiseau, France; Institute of Cell Biology, University of Edinburgh, EH9 3FF, Edinburgh, United Kingdom; Centre for Engineering Biology, University of Edinburgh, EH9 3BF, Edinburgh, United Kingdom; Institute of Cell Biology, University of Edinburgh, EH9 3FF, Edinburgh, United Kingdom; Centre for Engineering Biology, University of Edinburgh, EH9 3BF, Edinburgh, United Kingdom; Deanery of Biomedical Sciences, University of Edinburgh, EH8 9XD, Edinburgh, United Kingdom; Institute of Cell Biology, University of Edinburgh, EH9 3FF, Edinburgh, United Kingdom; Laboratory of Genome Integrity, National Cancer Institute (NCI), National Institutes of Health (NIH), Bethesda, MD20892-4254, United States; Biological Physics Research Group, Kavli Institute for Nanoscience Research, Department of Physics, University of Oxford, OX1 3QU, Oxford, United Kingdom; Biological Physics Research Group, Kavli Institute for Nanoscience Research, Department of Physics, University of Oxford, OX1 3QU, Oxford, United Kingdom; Institute of Cell Biology, University of Edinburgh, EH9 3FF, Edinburgh, United Kingdom; Centre for Engineering Biology, University of Edinburgh, EH9 3BF, Edinburgh, United Kingdom

## Abstract

Efficient DNA repair is essential for maintaining genome integrity and ensuring cell survival. In *Escherichia coli*, RecBCD plays a crucial role in processing DNA ends, following a DNA double-strand break (DSB), to initiate repair. While RecBCD has been extensively studied *in vitro*, less is known about how it contributes to rapid and efficient repair in living bacteria. Here, we use single-molecule microscopy to investigate DNA repair in real time in *E. coli*. We quantify RecB single-molecule mobility and monitor the induction of the DNA damage response (SOS response) in individual cells. We show that RecB binding to DNA ends caused by endogenous processes leads to efficient repair without SOS induction. In contrast, repair is less efficient in the presence of exogenous damage or in a mutant strain with modified RecB activities, leading to high SOS induction. Our findings reveal how subtle alterations in RecB activity profoundly impact the efficiency of DNA repair in *E. coli*.

## Graphical Abstract

**Graphical Abstract:**
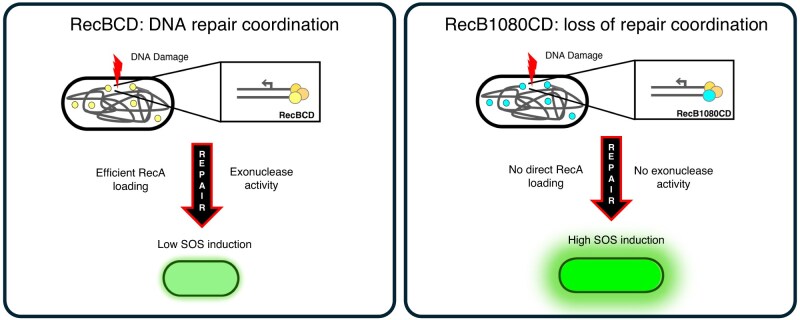

## Introduction

DNA repair is a fundamental mechanism that ensures chromosome maintenance and cell survival after DNA damage [1]. Among the different kinds of DNA lesions, DNA double-strand breaks (DSBs) are one of the most threatening to genome stability. Unrepaired DSBs can lead to cell death, while incomplete or faulty repair can induce mutagenesis and genome rearrangement [2]. DSBs can be caused by endogenous or exogenous causes, such as the collapse or stalling of replication forks, oxygen radicals, ionizing radiation, and DNA-damaging agents [3]. Quinolone antibiotics, which target DNA topoisomerases, disrupt DNA replication and induce DSBs, ultimately leading to bacterial cell death. This class of antibiotics, binding to the topoisomerase–DNA complex, interferes with the control of DNA supercoiling and causes the arrest of the replication machinery and the formation of DSBs [4]. However, DSBs can be repaired through homologous recombination, in which the missing information is copied from another intact, identical chromosomal copy [5].

In *Escherichiacoli*, the initial phase of the repair pathway involves the heterotrimer complex RecBCD [3] (Supplementary Fig. S1). This complex plays a crucial role in repairing DSBs by binding to DNA ends and processing them for subsequent homologous recombination. RecBCD is expressed at very low levels in cells [6] and the regulation of its expression levels is crucial for the cell’s DNA repair capability [7]. Although RecBCD expression is not upregulated by DNA damage, both deletion and overexpression of RecBCD strongly affect DNA repair, cell viability, and homologous recombination [8–10]. After it locates the DNA ends, RecBCD utilizes its two helicase motors with distinct polarities, namely RecB with a 3′ → 5′ direction and RecD with a 5′ → 3′ direction, to translocate along both DNA strands. During this translocation process, RecB’s nuclease activity actively degrades both DNA strands until it encounters a specific octameric DNA sequence known as χ-site (5′-GCTGGTGG-3′). The recognition of the χ-site triggers a modulation in RecBCD’s biochemical activities, leading to a drastic reduction in RecB’s nuclease activity at the 3′ single-stranded DNA (ssDNA) region. This alteration facilitates the loading of the RecA protein onto the 3′ ssDNA tail by RecBCD, forming a RecA-ssDNA filament. The RecA filament then catalyses homology search and strand invasion, facilitating homologous recombination.

RecA binding to single-stranded DNA also triggers LexA autoproteolysis, which activates the SOS regulon genes, allowing *E. coli* to respond to and repair DNA damage [11]. The SOS regulon comprises ∼40 genes. Among the genes regulated by LexA are DNA repair genes, such as RecA, and inhibitors of cell division, e.g. SulA [11, 12]. Inhibition of cell division by SulA results in bacterial cells appearing elongated with an increased cell area in comparison to cells without DNA damage [13].

DNA replication is the main physiological source of endogenous DSBs. It has been reported that in *E. coli* 18% of cells experience spontaneous replication fork breakage that leads to the formation of double-stranded DNA (dsDNA) ends [14]. Specifically, when the replication fork is arrested, the newly synthesized strands anneal to form a Holliday junction (with a free dsDNA end) end, which is stabilized by the RuvAB complex. Physiological DSBs caused by replication fork reversal can be repaired either through the exonuclease activity of RecB or by RecA-dependent homologous recombination (Supplementary Fig. S2) [15]. If the replication fork is restarted via the RecA-independent pathway, the SOS response is not induced. In that case, RecBCD binds to the dsDNA end and degrades the DNA up to the RuvAB-bound Holliday junction. Replication can then restart through a PriA-dependent mechanism [15–17].

*In vitro* studies have not only demonstrated the crucial role of RecBCD activity in recognizing and processing damaged DNA ends but have also highlighted its significance in ensuring the successful formation of the RecA ssDNA filament [18–23]. The study of RecBCD crystal structure bound to a DNA hairpin allowed an understanding of how RecBCD interacts with DNA clarifying the fate of the 3′ ssDNA after the RecC domain recognizes the χ-site. While RecBCD keeps degrading the 5′ side and translocating on the DNA, RecA is recruited on the forming ssDNA loop [18]. Interestingly, RecA recruitment has been associated with the presence of the RecB nuclease domain [21, 22, 24]. RecBCD mutants in which the RecB nuclease function is inactivated fail to recruit RecA to the 3′ ssDNA [25–27]. In particular, the *recBD1080A* mutant (known as *recB1080*) contains a single point mutation that inactivates the nuclease domain. *In vitro* data show that RecB1080 is a functional helicase that unwinds DNA without degrading the 3′ ssDNA. However, it has been observed that, while it still recognizes χ sites, it does not promote RecA loading onto the ssDNA.

The precise mechanism by which RecBCD disengages from the DNA remains to be fully elucidated. *In vitro* observations have led to the formulation of a model [28], suggesting that the RecBCD dissociation process is initiated after recognition of the χ-site. According to this hypothesis, after χ-site recognition, RecBCD continues to unwind the DNA beyond the χ-site and then the subunits disengage from the DNA. In this model, the possible impact of the RecA filament formation on RecBCD-DNA dissociation is not taken into account. However, considering the role of the RecB nuclease domain in recruiting RecA to the ssDNA and the intricate topological shape of the RecBCD–DNA–RecA complex, it may play a role in RecBCD disengagement from the DNA.

While *in vitro* studies have laid the foundations of the mechanisms of the repair and the enzyme’s activities, *in vivo* observations allow a deeper understanding of how DSB repair happens in the complex environment of a living cell. RecA filament formation and activity under various DSB-inducing treatments have been monitored using several fluorescent fusions and labelling techniques [29–32]. In a recent live-cell study [33], the observation of the disappearance of fluorescent loci placed on the DNA after DSB induction confirmed that, in *E. coli*, RecBCD exhibits high translocating speed on DNA (up to ∼1.6 kb/s) and high degradation activity (∼100 kb). Although recent work explored RecB mobility after mitomycin C treatment [34], it is still unclear how RecBCD dynamics change in response to various levels of DNA damage and how this correlates to the induction of the SOS response. Understanding RecBCD dynamics at different levels of DNA damage is crucial to reveal how different amounts of DSBs are detected and processed *in vivo*.

To characterize RecBCD-mediated DSB repair *in vivo*, we observed and quantified the mobility of single RecB molecules in live *E. coli* in real time. RecB has a crucial role in RecBCD functionality since it is the only subunit of the complex that acts as both nuclease and helicase, and as such, it is an excellent candidate to study RecBCD activities. We induced different levels of DNA damage using a fluoroquinolone antibiotic, ciprofloxacin, while monitoring the SOS response. We quantified how RecB mobility changes at different DNA damage levels. We identified three sub-populations of RecB molecules, corresponding to different molecular behaviour within the cells. Furthermore, we determined that the fraction of RecB molecules involved in the repair process is proportional to the level of DNA damage. To explore the impact on DSB repair when RecBCD cannot promote RecA filament formation, we quantified the mobility and the SOS induction in the *recB1080* mutant. Our observations are consistent with a model based on previous *in vitro* observations [27], which suggests an alternative pathway for loading RecA onto single-stranded DNA when RecB-mediated RecA loading is impaired. This implies that *in vivo*, the alternative repair pathway operates on a longer timescale and with reduced efficiency compared to the repair process in the wild type (WT).

## Materials and methods

### Strains and plasmid construction

*Escherichia coli* MG1655 strain and its derivatives were used in this study. The characteristics of all the strains and plasmids employed are described in Table 1. The construction of the strain carrying the RecB-HaloTag fusion (MEK65) has been previously described [6]. To build the strains containing the GFP expression reporter (MEK707 and MEK2324), *PsulA-mGFP* was cloned into a pOSIP plasmid [35] and integrated at a chromosomal locus into the genome by clone integration [35]. The construction of the pOSIP plasmid containing the fluorescent reporter (pSJR036) has been previously described [36]. After construction, the MEK707 and MEK2324 strains were checked by polymerase chain reaction (PCR) amplification of the insertion (see Supplementary Table S1).

**Table 1.** List of strains and plasmids used in this work

Name	Characteristics	Reference
*Bacterial strain*		
MG1655	*F-lambda-rph-1*	Lab stock
MEK65	MG1655, *recB::halotag*	[6]
MEK445	MG1655, *HK022::psfiA-mGFP*	[36]
MEK707	MG1655, *recB::halotag HK022::psfiA-mGFP*	This work
MEK716	MG1655, *recB1080:halotag*	This work
DL654	MG1655, *ΔrecA:CmR*	[39]
MEK1326	MG1655, *ΔrecB*	[7]
MEK2324	MG1655, *recB1080::halotag HK022::psfiA-GFP*	This work
MEK2033	MG1655, *ΔmotA::KnR HK022:PsulA-mGFP P21:Ptet01-mKate2*	[36]
MEK2628	MG1655, *pBAD::halotag*	This work
MEK2833	MEK2033 *recB1080*	This work
*Plasmids*		
pSJR036	*PsulA-mGFP* insertion by clone integration	[36]
pDL4174	pTOF with *recB1080*	This work, gift from the Leach Lab
pSF1	Expression of HaloTag under the control of the pBAD promoter	[6]

To image the HaloTag protein alone, the HaloTag was expressed under the control of an arabinose inducible promoter [6], in the strain MEK2628.

The *recB1080* mutation was introduced into the *recB-HaloTag* strain to create the *recB1080-HaloTag* strain (MEK716). This was achieved through plasmid-mediated gene replacement using a plasmid derived from pTOF24, pDL4174 [37]. The *recB1080* fragment was generated by PCR with the primers listed in Supplementary Table S1. A digestion site, HaeIII, was incorporated into the *recB1080* fragment to facilitate PCR verification of successful construction. Subsequently, the *recB1080* fragment was ligated into a pTOF24 backbone after the plasmid was digested at the PstI and SalI sites, resulting in pDL4174. After construction, MEK716 was checked by restriction digestion with HaeIII enzyme and PCR amplification.

Strains MEK2033 and MEK2833 used in the mother machine experiments were engineered to contain two reporters: *PsulA-mGFP*, described above, and *PtetO1-mKate2*, a constitutive expression reporter. Additionally, both strains have deletions of the *motA* gene to facilitate imaging in a mother machine microfluidic device [36, 38]. Strain MEK2833 carries an additional mutation, *recB1080*, which was introduced and validated following the protocols used to build strain MEK716. The DL654 [39] and MEK1326 strains have been used as control in the antibiotic susceptibility tests.

The sequences of the oligonucleotides used are listed in Supplementary Table S1.

### Antibiotic susceptibility tests

Antibiotic susceptibility tests were conducted by cultivating the relevant bacterial strains (MG1655, MEK65, MEK707, MEK716, MEK2324, MEK1326, and DL654) overnight in LB media at 37^○^C. From the overnight cultures, serial dilutions were prepared with a dilution factor of 10^−5^ starting from OD_600_ = 1, and each strain was subsequently plated using a 42-pinner onto LB agar plates containing varying concentrations of ciprofloxacin (0, 4, 10, 14, 16, and 20 ng/ml). These plates were then incubated overnight at 37^○^C (see Supplementary Fig. S8).

### Culture conditions and Halo labelling

For all microscopy-based experiments, cells were grown in M9 supplemented with 0.2% (w/v) of glucose, 2 mM MgSO_4_, 0.1 mM CaCl_2_, and 1× MEM Essential and MEM Non-Essential Amino Acids (Gibco^®^). For single-molecule labelling, we used the labelling protocol previously described [6] without the chemical fixation step. In brief, bacterial cultures from frozen −80^○^C stocks were grown with shaking (150 rpm) in the culture medium overnight (14–16 h) at 37^○^C. The overnight cultures were diluted (1:300) into 15 ml of medium and grown with shaking (150 rpm) at 37^○^C to the mid-exponential phase (optical density OD_600_ = 0.2–0.3). A volume of cells equivalent to 1 ml at OD_600_ = 0.2 was centrifuged and resuspended in 1 ml fresh medium supplemented with JF549 (Janelia Fluor^®^ HaloTag^®^ Ligands, Promega) at a final concentration of 1 μM. The culture was further incubated for 1 h at 37^○^C with shaking. After the labelling step, each sample was centrifuged for 3 min at 8000 rpm and the pellet was resuspended in 0.5–1 ml of the M9-based medium (dye-free). This washing step was repeated three to four times. At each step, cells were transferred to a new tube to facilitate the removal of the dye. After the last washing step, 2–2.5 μl of bacteria were added to an agar pad containing 2% agarose dissolved in M9 media.

To induce DNA damage, ciprofloxacin at the chosen concentration (4, 10, or 14 ng/ml) was added to the bacterial culture 150 min before microscopy. The same concentration was maintained in the agar pad during microscopy.

#### SYTOX labelling

The bacterial nucleoid of the *recB-HaloTag* strain MEK65, which does not carry the GFP SOS reporter, was labelled using SYTOX Green (Invitrogen) [40]. During the Halo labelling protocol, 500 nM of SYTOX Green was added 40–45 min before the beginning of the washing step.

### Microscopy set-up

Imaging was performed using an inverted microscope (Nikon Ti-E) equipped with an EMCCD Camera (iXion Ultra 897, Andor), a 100× TIRF Nikon objective (NA 1.49, oil immersion), and a 1.5× Nikon magnification lens (pixel size = 107 nm). Images were acquired via MetaMorph^®^ (Molecular Devices; v7.8.13.0) in Highly Inclined Laminar Optical (HILO) sheet [41] configuration. The HILO configuration was established using the iLas^®^ variable angle TIRF control window.

### Real-time DNA repair imaging

Fluorescence excitation was performed using 561 and 488 nm lasers (Coherent OBIS) and detected via a dual-wavelength dichroic filter (488/561 nm) (TRF59904, Chroma). This configuration was used to stimulate the fluorescent emissions from RecBHaloTag-JF549 and the fluorescent signal emitted by *PsulA-mGFP* reporter. Movies were acquired with continuous laser excitation at 561 nm at ∼15 mW with an exposure time of 12 ms (except for the MEK2628 strain, where the exposure time was 8 ms) for a total acquisition time of 7 s (600 frames). The camera’s electron-multiplying (EM) gain was set to 300, and the region of interest (ROI) was set to 256 × 256 pixels. A snapshot of the same ROI was acquired to image SOS induction by exciting the GFP signal with a 488 nm laser at ∼6 mW for 80 ms (camera EM gain 50). For each ROI, bright-field z-stacks of 16 images were acquired around the focus (total distance 3 μm, each step of 0.2 μm). Each bright-field image was acquired with 30 ms of exposure time and an EM camera gain of 4. When nucleoid images were acquired instead of the SOS induction signal, the SYTOX Green GFP signal was excited with the 488 nm laser at ∼6 mW for 30 ms (camera EM gain 4). Each sample was imaged for a maximum time of 40–45 min. All the acquisitions were performed at 37^○^C in an Okolab microscope cage incubator equipped with dark panels.

#### Cell segmentation

Cell segmentation was performed from bright-field images in BACMMAN [42], an ImageJ plug-in for high-throughput image analysis and manual curation. Bright-field images were first imported into BACMMAN as a ‘Dataset’. The ‘pre-processing’ step was then applied, which consisted of a single step that cropped the 16-image bright-field z-stack to 5 images on one side of the focus, as required by our cell segmentation algorithm. In the next step of the pipeline (the ‘processing’ step), cells were segmented using Talissman, a U-net-based segmentation algorithm (https://github.com/jeanollion/TaLiSSman). In brief, the U-net model predicts an Euclidean distance map, where the value of each pixel is its predicted distance to the nearest background pixel. A watershed algorithm is then applied to retrieve cell contours. This approach allowed us to accurately segment cells from bright-field images, including when they formed tight clusters. Following segmentation, post-filters were applied to dilate the segmented regions slightly (to make sure we would not miss any fluorescent spots located near the edge of the cell during single-particle tracking) and to remove any cells that were in contact with the edge of the image and might therefore be cropped. The resulting segmentation masks were finally exported in hdf5 format and imported to MATLAB to resolve cells during single-particle tracking.

#### Nucleoid detection

SYTOX Green fluorescence images were analysed in BACMMAN. First, a deep-learning-based denoising algorithm [43] was applied. Individual nucleoids were then segmented using a watershed algorithm on the maximum eigenvalues of the Hessian transform of the image. This approach allows precise segmentation of large spot-like objects with variable shapes. The segmented regions were exported in hdf5 format for further processing.

#### SOS induction signal detection and quantification

After performing bacterial cell segmentation, we computed the average fluorescent signal per cell area for each segmented cell in BACMMAN. The local fluorescent background was subtracted from each pixel of the image during the ‘pre-processing’ step using the ImageJ background subtraction method (Class: BackgroundSubstracted).

### Comparison of SOS induction in the WT and the RecB-HaloTag

All the data were acquired and analysed as described above, except for the data shown in Supplementary Fig. S4C and D, which were acquired and analysed as described below. Bacterial cells were grown in the same media described previously. Following overnight incubation, bacterial cultures were diluted (1:1000) into 15 ml of the medium and grown at 37^○^C until the OD_600_ reached 0.4. Ciprofloxacin was added when necessary at a concentration of 10 ng/ml, and the incubation continued for a total of 150 min. Samples were imaged on M9-based agar pads consisting of 2% agarose.

#### Fluorescent signal acquisition

Images were acquired on the same microscope set-up described above. The GFP signal was excited with a SpectraX Line engine (Lumencor) and a fluorescein isothiocyanate filter. The exposure time was 200 ms and the camera EM gain was 4. To identify cells within the ROI, we obtained seven bright-field z-stack images around the focal point, covering a total distance of 1.5 μm, with each step being 0.2 μm.

#### Image analysis

Bright-field z-stack and fluorescent images were analysed using the pipeline previously described [36]. In brief, bacterial cells in the ROI were segmented using an edge detection algorithm combined with a custom low-pass filter. The resulting cell outlines underwent manual curation to finalize the segmentation. The fluorescent signal was quantified within each cell, and the average fluorescent signal was calculated by averaging the total signal over the cell area. A local background was computed and subtracted from the average fluorescence, with the background being determined as the average fluorescent signal measured over an area located 15 pixels away from the cell border. Only bacterial cells that were at least 15 pixels away from other cells were included in the analysis, and other cells were excluded.

### Single-particle tracking

Single-particle tracking was performed using a custom-written script in MATLAB (MathWorks R2021a^®^). Single-particle localizations were identified by applying an intensity threshold and a bandpass filter to each frame of the video. The coordinates of each intensity peak centroid were computed using a Gaussian fit. Bacterial cell segmentation was used to associate the computed localizations with individual bacterial cells. Trajectories were built inside each segmented bacterium. Localizations within a tracking window of 5 pixels (0.53 μm) in successive frames were linked together to form a trajectory. In the case of multiple localizations in the tracking window, positions whose distance resulted in the minimal total squared displacement were associated with the same track.

The bandpass filter, peak-finder, and tracking functions are from previously developed and published software (http:// physics.georgetown.edu/matlab/) [44].

### Apparent diffusion coefficient calculation

The D* was calculated as in [45, 46] from the mean square displacement (MSD) of each trajectory divided by four times the time interval between frames, as


(1) \begin{eqnarray*}
D^{*}&=&\frac{1}{4 n \Delta t}\sum _{i=1}^{n}[x(i\Delta t+ \Delta t)- x(i\Delta t)]^{2}\nonumber\\ &&+\, [y(i\Delta t+ \Delta t)- y(i\Delta t)]^{2} ,
\end{eqnarray*}

where *x*(*t*) and *y*(*t*) are the trajectory’s position coordinates at time *t*, the camera exposure time is Δ*t*, and *n* is the number of frames. All trajectories were truncated at a fixed length of *n* = 4 frames (five localizations) to allow the comparison of D* values and use of analytical expression describing the distribution of D* (see below). D* distributions were obtained by combining all the D* values calculated for the entire sample. The distributions of the MSD and displacements are shown in Supplementary Fig. S3. The localization error was also taken into account and subtracted from the D* values [45].

#### Localization error

The average localization uncertainty in our experimental conditions was estimated using the Thunderstorm plug-in in Fiji [47] on three representative single-particle tracking datasets. The formula used for localization uncertainty is


(2) \begin{eqnarray*}
\langle (\Delta x)^2 \rangle = \frac{2 \sigma ^2 + a^2 / 12}{N} + \frac{8\pi \sigma ^4 b^2}{a^2 N^2} ,
\end{eqnarray*}

with *σ* the standard deviation of the fitted Gaussian in nm, *a* the pixel size in nm,*N* the number of detected photons, and *b* the background signal, evaluated as the residuals between the raw data and the fitted Gaussian. The obtained localization uncertainty of 28 nm was used with all datasets to compute the apparent diffusion coefficient.

### D* distribution fit

The probability of observing an apparent diffusion coefficient $D_i^*$ for an individual particle diffusing with D* and tracked over *n* frames is described by the following equation, as previously established [48]:


(3) \begin{eqnarray*}
p( D_{i}^{*})=\frac{1}{(n-1)!}\times \left({\frac{n}{D^*}}\right)^{n}\times [( D_{i}^{*})]^{(n-1)}\times \exp \left(\frac{-nD_{i}^{*}}{D^*}\right).
\end{eqnarray*}

As mentioned above, to compute D* for each trajectory, we consider trajectories of length *n* = 4 steps (five localizations). Therefore, to fit the D* histogram distributions, we used equation (3) for *n* = 4. For the *recB-HaloTag* strain not exposed to ciprofloxacin, we initially fit the D* distribution with a model describing two molecular species diffusing with $D_1^*$ and $D_2^*$:


(4) \begin{eqnarray*}
\begin{aligned} p( D_{i}^{*})=\frac{A}{6} \times \left({\frac{4}{D_1^*}}\right)^{4} \times [( D_{i}^{*})]^{(3)}\times \exp \left(\frac{-4D_{i}^{*}}{D_1^*}\right) \\ + \frac{1-A}{6} \times \left({\frac{4}{D_2^*}}\right)^{4} \times [( D_{i}^{*})]^{(3)}\times \exp \left(\frac{-4D_{i}^{*}}{D_2^*}\right) .\end{aligned}
\end{eqnarray*}

To describe the D* distribution after exposure to the antibiotic, we used a model describing three different sub-populations of molecules:


(5) \begin{eqnarray*}
\begin{aligned} p( D_{i}^{*})=\frac{A_1}{6} \times \left({\frac{4}{D_1^*}}\right)^{4} \times [( D_{i}^{*})]^{(3)}\times \exp \left(\frac{-4D_{i}^{*}}{D_1^*}\right) \\ + \frac{A_2}{6} \times \left({\frac{4}{D_2^*}}\right)^{4} \times [( D_{2}^{*})]^{(3)}\times \exp \left(\frac{-4D_{i}^{*}}{D_2^*}\right) \\ + \frac{1-A_1-A_2}{6} \times \left({\frac{4}{D_3^*}}\right)^{4} \times [( D_{i}^{*})]^{(3)}\times \exp \left(\frac{-4D_{i}^{*}}{D_1^*}\right) .\end{aligned}
\end{eqnarray*}

The three-sub-population fit was performed by constraining the value of the averaged D* of the very slow fraction of trajectories to 0.09 μm^2^/s. This specific value was determined by averaging the D* values associated with the slower sub-population, which were computed by fitting the D* distributions of the 14 ng/ml samples using a three-sub-population model for D*. Fits were performed using maximum likelihood estimation in MATLAB. Following the fitting calculation, individual trajectories can be assigned to one of the three distinct sub-populations.

### Mother machine experiments

#### Culture condition and sample preparation

The microfluidic chip’s design and microfabrication are described in [36, 38]. Bacteria were grown as described in the ‘Culture conditions and Halo labelling’ section. Briefly, the overnight culture was diluted (1:1000) in 50 ml of medium with a 0.01% (w/v) final concentration of Tween-20 Surfact-Amps detergent solution (Thermo Scientific, 85113) and grown until an OD_600_ = 0.2. Before loading in the chip, the sample was concentrated 100-fold by centrifugation (4000 rpm for 7 min). Before sample loading, the microfluidic device was passivated with the media with a 0.01% final concentration of Tween-20 Surfact-Amps. When ciprofloxacin was added, we used a final concentration of 3 ng/ml. The flow rate was controlled with a peristaltic pump (Ismatec IPC ISM932D) and set to 50 μl/min for the first 5 min and adjusted to 16 μl/min (1 ml/h) for the rest of the experiment. Bacteria were loaded in the flow channels with a 1-ml syringe and spun into the microchannels with a microcentrifuge (4000 rpm, 5 min) and a custom-built coverslip holder. The chips were mounted on the microscope with a custom-built chip holder. Bacteria were left in the chip for 2–4 h to adapt before starting imaging. The microfluidic device allowed for simultaneous comparison of two treatments per experiment: a no-antibiotic control and ciprofloxacin exposure at 3 ng/ml, applied to both the WT (MEK2033) strain and the mutant (MEK2833). The experimental protocol consisted of three phases: (i) an initial 4 h and 30 min period where all channels received medium without antibiotic, allowing cells to acclimate to the system and reach balanced exponential growth; (ii) a 5 h and 30 min exposure to antibiotic; and (iii) a final recovery phase of 3 h in medium without antibiotic. Since we measured that it takes ∼35–40 min for the antibiotic to fully reach the bacteria in the device channel, we consider the start of antibiotic exposure to be after this time.

#### Imaging and data acquisition

Images were acquired using the microscope set-up described in the ‘Microscopy set-up’ section, with control provided by MicroManager [49] through a MATLAB user interface [36]. The fluorescent signals emitted by the constitutive reporters *PsulA-mGFP* and *Ptet01-mKate2* were excited using a SpectraX Line engine (Lumencor). Specific filter sets were employed to detect these signals. For *PsulA-mGFP*, the filters used were ET480/40X (excitation), T510LPXR (dichroic), and ET535/50m (emission). In contrast, for *Ptet01-mKate2*, the filters used were ET545/30X (excitation), T590LPXR (dichroic), and ET632/60m (emission). All filters were purchased from Chroma. The exposure times for image acquisition were set as follows: bright-field images were acquired at 20 ms, GFP images at 80 ms, and mKate2 images at 100 ms. Images were acquired at 5-min intervals.

#### Image analysis and data analysis

As described in the ‘Cell segmentation’ section, cell segmentation and lineage tracking were performed using BACMMAN run in ImageJ [42]. The BACMMAN configuration was adapted to segment and track cells based on fluorescence images. Further data analysis was performed as described in [38].

### Calculation of the percentage of bacteria with high SOS induction

We assessed the percentage of cells exhibiting high SOS levels by considering those with SOS values exceeding 2 × 10^2^ arbitrary units (a.u.). This threshold was chosen based on the sub-population of bacteria expressing high SOS in the *recB-HaloTag* strain that was not exposed to ciprofloxacin (see Supplementary Fig. S9D).

### Statistical analysis

Three (or four) independent datasets have been acquired for each DNA damage condition. The results of the D* fits are presented as mean ± standard deviation (SD) computed over the three independent datasets.

### Code availability

BACMMAN is a plug-in in ImageJ (Fiji), and it can be installed following the instructions on BACMMAN GitHub. The MATLAB code used to perform the data analysis can be found in the MEKlab Gitlab.

## Results

### *In vivo* tracking of single RecB molecules using HaloTag labelling

To understand how DNA DSBs are recognized and processed by RecBCD, we measured the mobility of single RecB molecules comparing endogenous conditions and different levels of DNA damage. We used a previously characterized translational fusion of the HaloTag to RecB, having already established that the labelling is specific and the fusion can be used to image single RecB molecules that are functional [6].

To further test whether the HaloTag fusion influenced the downstream molecular processes of *E. coli* at the single-cell level, we compared the induction of the SOS response in two strains: the WT *E. coli* strain and the *E. coli* strain that contained the RecB-HaloTag fusion. Tests conducted at the single-cell level can detect effects that might be neglected when analysing the impact on DNA repair in *E. coli* in whole populations of bacteria [50]. We measured the cell area and evaluated the induction of the SOS response by calculating the mean GFP intensity per bacterial cell using an SOS transcriptional reporter, *PsulA-mGFP* [36]. This analysis was performed on both the WT *recB* (MEK445) and *recB-HaloTag* strains (MEK707) after incubating both strains with 10 ng/ml of ciprofloxacin for 2 h. As expected, after exposure to ciprofloxacin, we observed an increase in the cell area and induction of the SOS response (Supplementary Fig. S4A and C). The cell area and the SOS signal distributions of the *recB-HaloTag* strain were similar to those measured for the WT *recB* (Supplementary Fig. S4B and D). Thus, our data show that the HaloTag fusion does not affect the capacity of RecBCD to lead to the induction of the SOS response, further confirming that the *recB-HaloTag* strain can be used to study DNA DSB repair in live *E. coli*.

**Figure 1.**
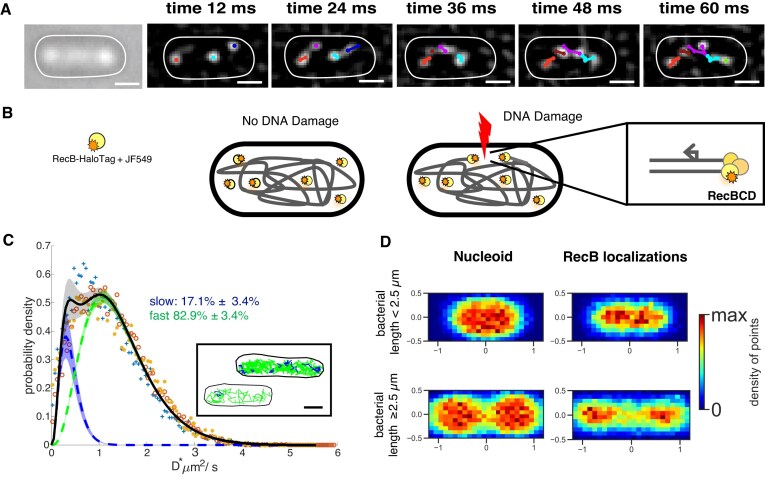
Single RecB molecule tracking in live bacteria. (**A**) Illustration of RecB single-molecule trajectories detected in a single bacterial cell for five consecutive frames (see also Supplementary Fig. S5A). Left panel: bright-field. Right panels: progression of the track building overlapped on filtered images to highlight diffraction-limited spots of the frame corresponding to the indicated time (top of each panel). Scale bar: 1 µm. (**B**) Schematic of RecB-HaloTag mobility labelled with JF549. In the absence of DNA damage (on the left), RecB-HaloTag mainly undergoes free diffusion. Following DNA damage (on the right), RecB-HaloTag binds to DSB ends. (**C**) Apparent diffusion coefficients distribution, D*, of the detected RecB single-molecule trajectories for three datasets [total number of bacteria: 2830; total number of tracks: 25 134; dataset 1: ‘+’, dataset 2: ‘o’ , dataset 3: ‘⋆’ ; see Supplementary Table S2 for information on single datasets]. The averaged fitted distribution describing two sub-populations of RecB trajectories with different mobility is overlapped (full black line). Dotted lines represent the averaged fitted curves, while shaded areas denote the SD from the average of the fits conducted on each dataset. Fractions of trajectories described by each sub-population are indicated. Inset: representative examples of RecB detected trajectories colour-coded as the respective D* sub-population. In blue are the trajectories whose D* is associated with the slower sub-population, and in green are the ones whose D* is associated with the faster sub-population. Scale bar: 1 μm. (**D**) Localization maps of RecB molecules in bacterial cells and nucleoids' spatial distribution for cell length smaller (top panels) and equal to or longer than 2.5 µm (bottom panels), each normalized by bacterial cell length and width. Nucleoids, *N*_cells_ = 124; RecB localizations, *N*_cells_ = 1127.

The HaloTag conjugated with the synthetic dye JF549 [51] enables *in vivo* tracking of rapidly diffusing proteins [52]. Combined with RecB low copy number (on average 4.9 ± 0.3 [6]), it allowed us to directly track single RecB molecules without needing photoactivation imaging techniques (Fig. 1A and B).

To estimate the mobility of a single RecB trajectory, we computed its apparent diffusion coefficient, D*, as previously described [46, 52–55]. We first computed the D* distribution of single RecB molecules in cells not exposed to exogenous sources of DNA damage. We initially performed a fit of the D* histograms using an analytical expression of D* [48] representing one diffusing population of molecules (Supplementary Fig. S5B–D). The value of the fitted D* averaged over the values computed for each of the three datasets was 1.22 ± 0.10 μm^2^/s (Supplementary Fig. S5B–D). However, we noticed that the one-population fitted distributions shown in Supplementary Fig. S5 failed to fully describe the underlying D* histogram, prompting us to use a two-population fit.

The two-sub-population fit (Fig. 1C) identified a first group of RecB trajectory described by an average D* = 0.40 ± 0.02 μm^2^/s and a second one described by an average D* = 1.43 ± 0.05 μm^2^/s (as above, the D* values were averaged from fits performed on three datasets acquired in the same conditions; Supplementary Fig. S5E–G and Supplementary Table S3). The majority of RecB trajectories (82.9% ± 3.4%, Supplementary Table S3) showed high mobility (D* = 1.43 ± 0.05 μm^2^/s). This population of RecB trajectories likely corresponds to RecB molecules that are diffusing in the cytoplasm and do not interact with the DNA. The second population of RecB trajectories, described by the average D* = 0.40 ± 0.02 μm^2^/s, corresponded to 17.1% ± 3.4% of the entire population. As observed in other DNA-interacting proteins under similar imaging conditions [46], the mobility of this fraction of RecB trajectories is too high to be attributed to molecules bound to the DNA. Thus, it is likely that this subset of RecB trajectories corresponds to a sub-population of molecules engaged in transient interactions with DNA as they search for their target sites although we cannot exclude that some of those molecules appear in the slow sub-population as a result of confinement. This two-population fit does not take into account very slow RecB trajectories corresponding to RecB bound to DNA in line with the low frequency of endogenous DSBs [14].

To further verify that the RecB D* distribution represents the mobility of RecB molecules and their interaction with DNA, we quantified the mobility of the HaloTag alone and confirmed that it followed a different D* distribution. We observed that this protein diffused freely within the bacterial cell, showing a higher D* than RecB in line with its smaller molecular weight (35 kDa). Importantly, it did not show a sub-population corresponding to a slow D*, as expected from a protein that cannot interact with DNA (Supplementary Note S1 and Supplementary Fig. S6).

We verified that RecB molecules are localized mainly within the bacterial nucleoid, by building a two-dimensional localization map of the detected RecB molecules for all the bacterial cells of our samples (see Fig. 1D, right panel, and Supplementary Fig. S7 for more datasets). We then used the SYTOX Green dye [40, 46] (see also the ‘Materials and methods’ section) to label and image the bacterial nucleoid. Comparing the nucleoid positions to the RecB localization distribution map (Fig. 1D, left panel), we observed that, as expected, the RecB spatial distribution overlapped with the spatial distribution of the nucleoid. For larger cells where the chromosome has started to segregate prior to cell division (i.e. bacterial cells longer than 2.5 μm) and forms a typical bi-lobar shape, the localization of RecB molecules showed a very similar shape indicating that they likely co-localize with the bacterial nucleoid during the cell cycle (see Supplementary Fig. S7F).

### RecB mobility decreases with a high level of induced DNA damage

To investigate how different levels of DNA damage could impact the repair process and RecB mobility, we treated bacterial cells with sub-lethal concentrations of ciprofloxacin. We aimed to identify concentrations of ciprofloxacin that would induce the SOS response without leading to cell death. We used spot test assays across a range of ciprofloxacin concentrations from 0 to 20 ng/ml (see the ‘Materials and methods’ section and Supplementary Fig. S8). We chose 4 and 14 ng/ml for the following reasons: at 4 ng/ml, low-level DNA damage is produced but viability is not affected (Supplementary Figs S8 and S17); at 14 ng/ml, the level of DNA damage is higher but the spot tests showed a limited reduction in cell viability (see Supplementary Fig. S8), thus allowing us to observe the repair process. We quantified the average GFP signal per area from the SOS fluorescent reporter *PsulA-mGFP* at the single cell level and measured the cell area in these conditions. Bacteria were exposed to each concentration of ciprofloxacin for 150 min before starting microscopy and the same antibiotic concentration was maintained on the agar pad, thus ensuring that the cells reached a ‘steady state’ of DNA damage exposure. We verified that these two concentrations led to increasing levels of SOS induction (Supplementary Fig. S9B and D): of note, based on these data, without exposure to ciprofloxacin, 3% of cells induce SOS, as previously observed [56, 57].

**Figure 2.**
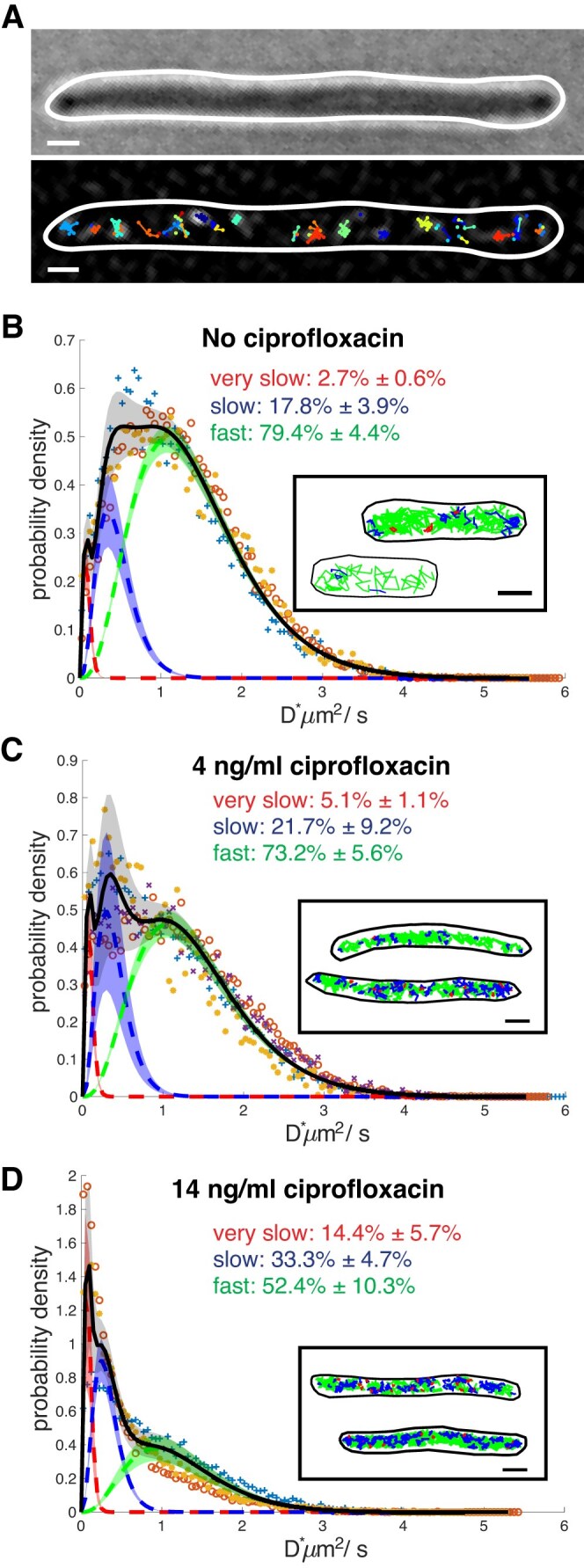
RecB mobility decreases with a high level of DNA damage. (**A**) Representative example of RecB trajectories detected in a cell exposed to 14 ng/ml of ciprofloxacin. Top panel: bright-field. Bottom panel: detected trajectories overlapped on one filtered image. Scale bar: 1 µm. Apparent diffusion coefficient distributions, D*, for samples exposed to (**B**) no ciprofloxacin [same datasets shown in Fig. 1 here fitted with a three-sub-population model, dataset 1: ‘+’, dataset 2: ‘o’, dataset 3: ‘⋆’ ], (**C**) 4 ng/ml of ciprofloxacin [dataset 1: ‘+’, dataset 2: ‘o’, dataset 3: ‘⋆’, dataset 4: ‘x’], and (**D**) 14 ng/ml of ciprofloxacin [dataset 1: ‘+’, dataset 2: ‘o’, dataset 3: ‘⋆’ ]. Histograms were fitted with a three-species model (full black line) corresponding to three sub-populations of very slow (dotted red line), slow (dotted blue line), and fast (dotted green line) moving RecB molecules. Dotted lines are averaged fitted values, and shadow areas represent the SD computed from the datasets acquired in each condition. Fractions of trajectories described by each sub-population are indicated. Insets: representative examples of RecB detected trajectories for the corresponding condition, colour-coded as the respective D* sub-population. In red the trajectories described by $D_{{\rm very\,slow}}^{*}$, in blue the trajectories described by $D_{{\rm slow}}^{*}$, and in green the trajectories described by $D_{{\rm fast}}^{*}$. Scale bar: 1 μm.

We performed RecB single-molecule tracking under ciprofloxacin exposure. As expected, following ciprofloxacin treatment, the bacterial cells appeared elongated (Supplementary Fig. S9A and C), and we observed that the detected RecB trajectories (Fig. 2A) explored a smaller space compared to the condition without ciprofloxacin (Fig. 1A). This suggests that some RecB molecules were recruited to the DNA and, hence, appeared much less mobile. Indeed, in the presence of DSBs, under our imaging conditions, we expected to detect very slow or almost immobile RecB molecules, indicating that RecBCD is recruited to the DNA double-strand ends (Fig. 1B), in keeping with very slow diffusion of the DNA polymer [58]. While RecBCD translocates along DNA, our spatiotemporal resolution limits our ability to measure translocation directly.

The D* distributions for 4 and 14 ng/ml of ciprofloxacin (Fig. 2C and D) showed a clear shift towards values of D* smaller than 1 μm^2^/s in comparison to the D* distribution computed for the no ciprofloxacin sample. We also noticed a peak (more evident in the 14 ng/ml of ciprofloxacin condition) in the D* distribution for D* values lower than ∼0.10 μm^2^/s (Fig. 2C and D). Similar values of D* have been previously associated with molecules bound to the DNA [45, 46, 54, 59]. Therefore, we fitted the D* histograms of trajectories obtained in the presence of 14 ng/ml ciprofloxacin with an analytical expression of D* containing a third, additional sub-population of RecB trajectories (see Fig. 2, Supplementary Figs S10 and S11A–C, and Supplementary Table S4). The fit was performed by constraining the value of the averaged D* of the very slow fraction of trajectories to 0.09 μm^2^/s (see the ‘Materials and methods’ section). The fit provided an estimate for the relative proportions of each sub-population of RecB trajectories: 14.4% ± 5.7% of the detected trajectories were ‘very slow’; 33.3% ± 4.7% were ‘slow’ ($D_{{\rm slow}}^{*}$ = 0.33 ± 0.02 μm^2^/s); and the remaining 52.4% ± 10.3% were ‘fast’ ($D_{{\rm fast}}^{*}$ = 1.24 ± 0.07 μm^2^/s). To quantify how the relative fraction of RecB trajectories in the sub-populations changed for the different levels of DNA damage, we performed the same fit for the D* distribution computed for the bacteria exposed to no and 4 ng/ml of ciprofloxacin (see Supplementary Table S4 and Supplementary Figs S10 and S11A–C). We observed that the fraction of trajectories corresponding to $D_{{\rm very\,slow}}^{*}$ progressively increased with the level of DNA damage from 2.7% ± 0.6% without ciprofloxacin to 5.1% ± 1.1% at 4 ng/ml of ciprofloxacin to reach 14.4% ± 5.7% at 14 ng/ml of ciprofloxacin. The fraction of RecB trajectories corresponding to the fast sub-population did not vary significantly between 0 and 4 ng/ml of ciprofloxacin with values between 79.4% ± 4.3% and 73.2% ± 5.6% respectively, but it decreased for the highest level of DNA damage to 52.4% ± 10.3% (see Supplementary Figs S10 and S11A–C, and Supplementary Table S4). The reduction in the fraction of fast (non-DNA interacting) molecules for the higher concentration of ciprofloxacin was consistent with an increase of RecB molecules engaged in the repair process. Collectively, these results suggest RecB mobility experiences small variations at lower concentrations of ciprofloxacin, whereas it is significantly affected at a high ciprofloxacin concentration. This reflects the expected increase in the number of DSBs correlated with increasing ciprofloxacin concentrations.

### RecB nuclease inactivation changes the dynamics of its interaction with DNA

To investigate how DSBs are processed when the RecBCD-dependent repair pathway is affected, we chose to observe RecB activity and SOS induction in the presence of a mutated RecB protein, RecB1080. This protein carries a single point mutation in the putative Mg^2+^ binding site of the RecB subunit (Asp-1080 → Ala), which inactivates the RecB nuclease domain [25, 26]. Biochemical analysis of RecB1080CD shows that the complex still recognizes χ sites but does not promote RecA loading [26]. Hence, it is not able to complete DSB repair through the usual RecBCD-dependent RecA loading pathway, although it is still partially functional since the helicase activities are not affected.

We constructed a HaloTag fusion to the mutated RecB subunit and introduced the SOS transcriptional reporter *PsulA-mGFP* into the mutant chromosome (MEK2324), as previously performed for the *recB-HaloTag* strain. Characterization of this strain (referred to as the *recB1080-HaloTag* mutant) showed normal viability (Supplementary Figs S12A, top panel, and S17) although some cells were elongated (Supplementary Fig. S12A, bottom panel). Survival after exposure to various concentrations of ciprofloxacin (4 and 14 ng/ml; see Supplementary Fig. S12A, top panel), while it reduced compared to WT, was much higher than in a Δ*recB* strain. This is similar to previous results obtained after exposure to gamma irradiation [27] and suggests that the *recB1080-HaloTag* mutant is able to repair DSBs by loading RecA through another, less efficient, pathway (described in Supplementary Fig. S13 [27]). Hence, this mutant allowed us to measure the efficiency of DNA repair when the main RecA loading pathway is inactivated without confounding factors linked to the low viability of most *recBCD* mutants.

**Figure 3.**
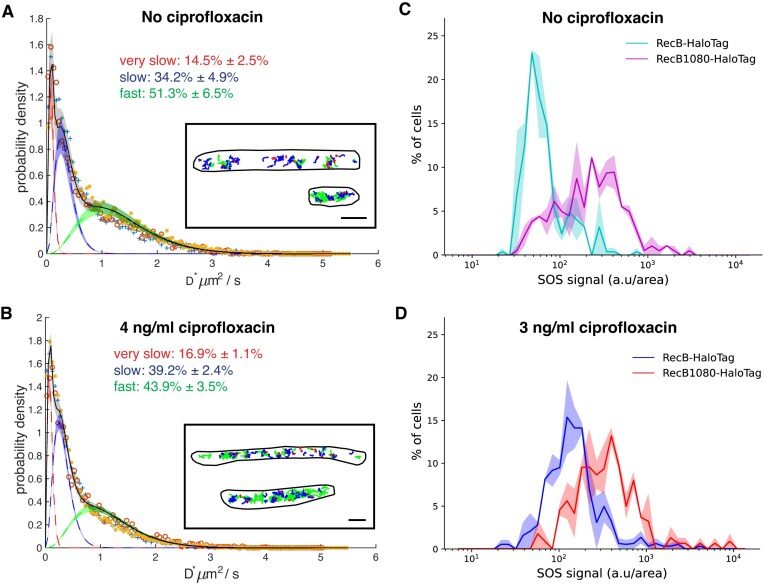
The DNA repair dynamics is altered in *recB1080-HaloTag* mutant. Apparent diffusion coefficient distributions, D*, for samples treated with (**A**) no ciprofloxacin [total number of bacteria: 1232; total number of tracks: 16 444; dataset 1: ‘+’, dataset 2: ‘o’, dataset 3: ‘⋆’; see Supplementary Table S5 for information on single datasets] and (**B**) 4 ng/ml of ciprofloxacin [total number of bacteria: 1132; total number of tracks: 26 861; dataset 1: ‘+’, dataset 2: ‘o’, dataset 3: ‘⋆’; see Supplementary Table S5 for information on single datasets]. The averaged fitted distribution describing three sub-populations of RecB trajectories with different mobility is overlapped (full black line). Dotted lines are averaged fitted values and shadow areas represent the standard deviation computed from the datasets acquired in each condition. Fractions of trajectories described by each sub-population are indicated. Insets: representative examples of RecB1080 detected trajectories for the corresponding condition, colour-coded as the respective D* sub-population. In red trajectories corresponding to $D_{{\rm very\,slow}}^{*}$, in blue trajectories corresponding to $D_{{\rm slow}}^{*}$, and in green trajectories corresponding to $D_{{\rm fast}}^{*}$. Scale bar: 1 μm. (**C**) *recB1080-HaloTag* cells exhibit elevated SOS induction even without ciprofloxacin exposure. (**D**) Ciprofloxacin treatment increases SOS levels in both strains, with the *recB1080-HaloTag* showing higher SOS induction. Full lines represent the average computed over three datasets, and shaded areas represent the standard deviation.

To characterize the *in vivo* activity of RecB1080-HaloTag, we performed tracking of single RecB1080-HaloTag molecules. Without ciprofloxacin, the D* distribution of RecB1080-HaloTag was dramatically different from the one observed in the same condition for the RecB-HaloTag strain. Indeed, the fraction of very slow (D^*^= 0.09 μm^2^/s) RecB1080-HaloTag molecules was 14.5% ± 2.5%, five to six times larger than for RecB-HaloTag with 2.7% ± 0.6% (Fig. 3A, Supplementary Figs S11D and S12B–D, and Supplementary Table S6). The fraction of slow RecB1080-HaloTag molecules (D* = 0.34 ± 0.2 μm^2^/s) was 34.2% ± 4.9% and the third population of fast RecB1080-HaloTag molecules (D^*^= 1.33 ± 0.03 μm^2^/s) was 51.3% ± 6.5%. Given that the number of DSB created per chromosome is not expected to change in the mutant, the higher fraction of very slow RecB1080 molecules suggests that RecB1080 molecules could stay bound to DNA ends for a longer time than the WT RecB, resulting in a shift in the proportion of different types of molecule mobility.

After exposure to 4 ng/ml of ciprofloxacin, we observed that the fraction of very slow RecB1080-HaloTag molecules increased to 16.9% ± 1.1% from 14.5% ± 2.5%. The fraction of slow RecB molecules (D^*^= 0.32 μm^2^/s) was 39.2% ± 2.4% and the third population of fast RecB molecules (D* = 1.15 ± 0.05 μm^2^/s) was 44.0% ± 3.5% (Fig. 3B and Supplementary Figs S11E and S12E–G). The increase in the proportion of very slow molecules is similar to WT-RecB cells at this concentration and is consistent with the low levels of DNA damage caused by this concentration of ciprofloxacin. This suggests that the amount of exogenous DNA damage induced in *recB1080* is likely to be similar to WT, although the dynamic of the repair process might be altered.

### SOS induction is altered upon RecB nuclease inactivation

To further understand DNA repair dynamics, we compared two parameters—the fraction of cells with very slow RecB and the level of SOS induction—imaged simultaneously in individual cells. We calculated the proportion of cells with at least one very slow RecB in the WT and in the *recB1080* strains as well as the proportion of cells that had strongly induced SOS (Table 2). For endogenous DNA damage, in the *recB-HaloTag* strain, ∼12% of cells showed at least one RecB bound to DNA but only 2.8% of the cells had induced high SOS levels, suggesting that DNA repair is efficient and rarely leads to full SOS induction in keeping with previous results [56, 57]. In contrast, in the *recB1080-HaloTag* mutant, the proportion of cells with at least one DNA-bound RecB was very high (46.6%) with a similarly high number of cells (38.6%) that had induced SOS. This high proportion of cells with DNA-bound RecB1080 is likely due to a combination of two phenomena: first, as shown above, RecB1080 may stay bound to DNA for a longer time than WT RecB. Second, as an induced SOS state results in a larger cell volume and more DNA per cell [60], this could lead to a larger number of DSBs per cell. Upon exposure to ciprofloxacin, we observed a simultaneous rise in the proportion of cells with RecB bound to DNA (28.8% in WT and 67.5% in *recB1080-HaloTag*) and exhibiting high SOS induction as expected (59.5% and 50.8%, respectively, Table 2).

**Table 2.** Percentage of bacteria with RecB on the DNA and high SOS induction for the *recB-HaloTag* and *recB1080-HaloTag* strains

Strain	Ciprofloxacin (ng/ml)	RecB on the DNA substrate (% of bacteria)	High SOS induction (% of bacteria)
*recB-HaloTag*	0	12.3 ± 2.8	2.8 ± 0.4
	4	28.8 ± 9.0	59.5 ± 13.8
*recB1080-HaloTag*	0	46.6 ± 12.4	38.6 ± 10.7
	4	67.5 ± 14.2	50.8 ± 3.2

We then followed the SOS induction in real time in the WT and the *recB1080* strains using a mother machine microfluidic device [61] (Supplementary Fig. S14). Initial quantification of the distribution showed, as expected from our previous observations, that even without exposure to ciprofloxacin, the SOS signal distribution in the *recB1080* mutant was very different from that in the WT strain: a large number of cells had induced a detectable level of SOS (Fig. 3C). After 150 min of exposure to a ciprofloxacin concentration of 3 ng/ml, both the *recB1080* and the WT strains showed an increase in bacterial cells with high SOS expression (Fig. 3D). Notably, the *recB1080* mutant exhibited higher levels of SOS induction than the WT strain, indicating that in this mutant, DNA damage triggers a response above the already high level observed in endogenous conditions.

We then observed the SOS induction dynamics over time by monitoring the SOS signal of bacteria in the mother machine for 13 h (Supplementary Fig. S15). After an initial adaptation period of 4 h and 30 min, we exposed the bacteria to 3 ng/ml of ciprofloxacin for 5 h and 30 min: we observed a rapid increase in SOS signal in both strains. Although the WT strain reached a steady-state level of SOS induction after ∼2 h, the *recB1080* strain’s SOS signal continued to rise. It appeared to reach a plateau only after 3 h and 30 min of exposure. Notably, while removal of ciprofloxacin resulted in WT SOS levels returning to pre-exposure levels, as previously reported by [36, 38], the *recB1080* strain failed to shut off its SOS response in our observation time frame, highlighting that inactivation of the nuclease activity in this mutant strongly impairs its repair efficiency. These results confirm that the *recB1080* strain exhibits altered SOS response dynamics compared to the WT strain, with increased SOS induction upon damage and slower recovery.

Our observations suggest that altering RecBCD activities in the *recB1080* profoundly affects the cells’ repair capacity. In the case of endogenous damage due to fork reversal, the lack of exonuclease activity precludes fork restoration through DNA degradation (as depicted in Supplementary Fig. S2, right pathway), making it necessary to repair all forks through RecA-dependent SOS induction (Supplementary Fig. S2, left pathway) and explaining the large number of SOS induced cells in this mutant. When exposed to ciprofloxacin, SOS is induced for a longer time and repair is less efficient. This is likely due to the reduced capacity of RecB1080 to load RecA and promote the subsequent repair steps. Taken together, these results suggest that the dynamics of the SOS induction and the repair timescale in the *recB1080-HaloTag* are altered compared to the WT *recB-HaloTag* strain, highlighting the importance of all RecB biochemical activities in the repair process.

## Discussion

### The three sub-populations of RecB molecules correspond to different modes of interaction with DNA

In this work, we used single-molecule tracking in live *E. coli* to achieve a quantitative understanding of the initial steps of DSB repair *in vivo*. We observed that RecB mobility patterns change depending on its engagement in the repair process and that more RecB molecules are recruited onto DNA as the level of DNA damage increases.

Our data show that RecB binding to DNA correlates with the presence of DNA double-strand ends. In the absence of exogenous DNA damage, ∼2.7% of RecB trajectories are very slow, likely corresponding to RecB bound to the DNA. We also observed that ∼20% of RecB trajectories (see Fig. 2B and Supplementary Table S4) are slow, suggesting transient interactions with DNA, probably corresponding to RecB molecules engaged in target search, with the rest freely diffusing in the cytoplasm. Interestingly, our observation of the slow sub-population of RecB molecules, which may transiently interact with DNA while searching for target sites, is supported by *in vitro* findings of RecBCD non-specific interaction with DNA [62].

Exposure to exogenous DNA damage results in a critical change in the distribution of RecB interactions with its substrate: a significant proportion of RecB molecules display very slow mobility corresponding most likely to DNA-bound molecules, and the fraction of RecB not involved in the repair process decreases as more molecules bind to DSBs. Our observations are compatible with previous studies of the mobility of DNA repair enzymes, such as PolI, LigA, and MutS [45, 59]. All these enzymes bind to damaged DNA, and exhibit a comparatively low fraction of molecules bound to DNA in normal conditions and an increase upon exposure to DNA damage. Similarly, recent work investigating RecB dynamics after exposure to mitomycin C showed an increase of molecules with very low mobility after DNA damage [34].

Our data can also be used to estimate the number of dsDNA ends, under the assumption that cells with no DNA-bound RecB do not have a DSB. This fraction can then be used as a parameter in a Poisson distribution assuming DSBs are formed in an independent process. The computed Poisson distribution (Supplementary Fig. S16) shows that in the absence of ciprofloxacin, ∼10% of the cells have one dsDNA end consistent with rates of spontaneous dsDNA ends formation measured previously [14]. At 4 ng/ml of ciprofloxacin, 24% of bacterial cells have one DSB end and 4% have two DSBs, consistent with the low level of DNA damage induced. At 14 ng/ml, most cells contain between one and five DSBs, indicating that ciprofloxacin induces multiple DSBs per cell. This may overwhelm repair mechanisms and lead to cell death, consistent with reduced population viability at this concentration (Supplementary Fig. S8).

Our observations are based on tracking the RecB subunit, most likely as part of the RecBC or RecBCD complexes that are both known to interact with DNA [3, 18, 63, 64]. Despite their distinct biochemical activities, it is not possible to distinguish them precisely via single-molecule tracking. Their apparent diffusion coefficients, when non-interacting with DNA, are expected to be nearly indistinguishable as a result of their high molecular weights (MW RecBC: 263 kDa; MW RecBCD: 330 kDa [46, 65]). Although RecBCD initiates repair from blunt ends much more efficiently than RecBC [66], they both robustly interact with DNA ends [64], making differentiation of the complexes challenging. It is also possible that we may have detected uncomplexed individual RecB subunits. We believe these would represent only a small number of the molecules because RecB forms a tight complex with RecC [18]. Moreover, their impact is likely confined to the fast fraction of the trajectories, as RecB alone lacks robust DNA binding activities [67], in contrast to RecBC and RecBCD.

### In the *recB1080* mutant, DSB repair dynamics differ from the WT

Combining RecB single-molecule tracking and the measurement of SOS induction in individual cells highlights key differences between the normal repair pathway and the alternative RecA loading mechanisms in the *recB1080* mutant. When WT RecB is produced, the repair of endogenous damage occurs efficiently without triggering a high SOS response. We observe 12% of the cells with a DNA-bound RecB molecule (see Table 2), suggesting that they have accumulated dsDNA ends, most likely from replication fork collapse [17] or from over-replication arising from converging replication forks at the terminus [68, 69]. In these conditions, it is likely that repair occurs through degradation of the reversed fork without SOS induction (Supplementary Fig. S2, right pathway). Alternatively, if repair occurs through recombination (Supplementary Fig. S2, left pathway), a homologous copy of the chromosome is close by, probably enabling efficient repair with a short ssDNA-RecA filament, which does not lead to a high SOS response. Indeed, the lifetime of RecA structures has been reported to be proportional to the induced SOS response [32].

In the *recB1080-HaloTag*, nearly 40% of bacterial cells have induced SOS, ∼20 times more than that observed in the WT strain (see Table 2) and similar to the proportion of bacterial cells that have at least one DNA-bound RecB1080. This suggests that RecB1080 binding to DSBs almost always results in SOS induction. This is likely due to this mutant’s lack of exonuclease activity, which triggers repair exclusively through a homologous recombination-dependent pathway (Supplementary Fig. S2, left pathway). Moreover, our results indicate that RecB1080 likely remains bound to DSBs longer than RecB. As illustrated in Supplementary Fig. S13, in the absence of its exonuclease activity, RecB1080 fails to degrade the DNA strands before χ recognition (although alternative exonucleases such as RecJ are likely providing some degradation activity): this might make dissociation from more difficult; alternatively, the lack of interaction with RecA or a very reduced capacity of RecA loading in this mutant might also affect its ability to dissociate. Last, we observed in this mutant a longer rise of SOS upon exposure to ciprofloxacin: this could be explained if loading of RecA using the alternative RecFOR pathway requires more time in the *recB1080-HaloTag* mutant compared to the *recB-HaloTag* strain. This is consistent with previous population-based measurements of SOS induction [70], which reported slower SOS induction in a *recB1080* mutant.

Our observations contribute to the *in vivo* understanding of DSB repair, providing valuable insights into the interaction between the RecBCD complex and DNA, as well as its ability to respond to varying levels of DNA damage. Our data concerning the *recB1080* mutant confirm the hypothesis that an alternative repair pathway is activated when RecB lacks its nuclease activity. By introducing a point mutation in the RecB nuclease domain to inactivate its exonuclease activity, we were able to understand how a relatively limited perturbation in RecBCD activities affects repair efficiency. Our results highlight the importance of the coordinated action of RecBCD helicase and nuclease activities, along with RecA loading, in achieving rapid and efficient repair. Moreover, such an experimental approach, using minimal, targeted perturbation of a highly coordinated process, could be used to probe other fundamental biological processes *in vivo*.

## Supplementary Material

gkaf454_Supplemental_File

## Data Availability

The data and the codes that support the findings of this study are available on BioImage Archive (number: S-BSST1968).
