# Supplementary material for: In vivo single-molecule imaging of RecB reveals efficient repair of DNA damage in Escherichia coli
Source: Nucleic Acids Res. 2025 Jun 4;53(10):gkaf454. doi: 10.1093/nar/gkaf454 (PMC12135197; doi:10.1093/nar/gkaf454)
Supplement: gkaf454_Supplemental_File [file gkaf454_supplemental_file.pdf]

Supplementary information

In vivo single-molecule imaging of RecB reveals efficient repair of DNA damage in *Escherichia coli*

Alessia Lepore [1,2,3 *], Daniel Thédié [1,2], Lorna McLaren [1,2], Louise Goossens[4], Benura Azeroglu [1,5], Oliver J. Pambos [6], Achillefs N. Kapanidis [6] and Meriem El Karoui [1,2, *]

[1]Institute of Cell Biology, University of Edinburgh, Edinburgh, UK; [2]Centre for Engineering Biology, University of Edinburgh, UK; [3]Laboratory for Optics and Biosciences, Ecole Polytechnique, Institut Polytechnique de Paris, Palaiseau, FR; [4]Deanery of Biomedical Sciences University of Edinburgh, UK; [5]Laboratory of Genome Integrity, National Cancer Institute (NCI), National Institutes of Health (NIH), Bethesda, MD, USA; [6]Biological Physics Research Group, Kavli Institute for Nanoscience Research, Department of Physics, University of Oxford, Oxford, UK

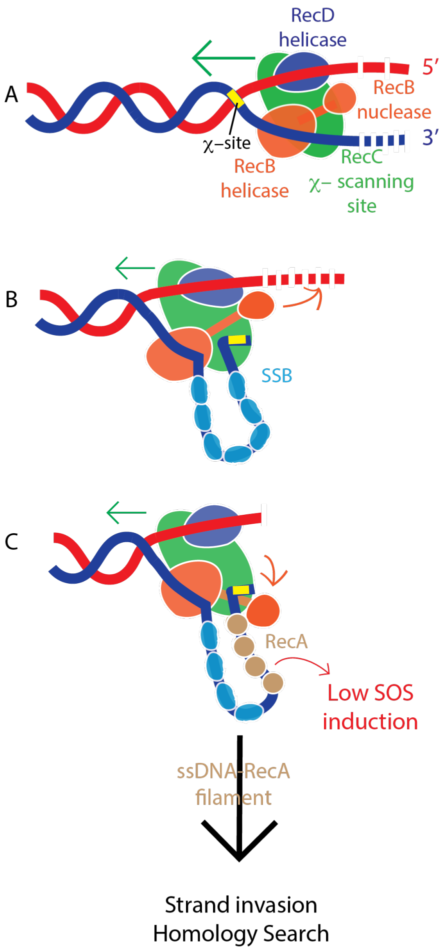

Supplementary Figure 1: Schematics of the RecBCD-mediated RecA loading repair pathway.
(A) RecBCD translocates on DNA while degrading it. **(B)** After χ– site recognition, RecBCD changes its biochemical activity. It pauses and then restarts translocation at a reduced rate; since the χ sequence is bound to the RecC subunit the 3' end exit is blocked. As a result, a loop of ssDNA is accumulated upstream of the χ– site and it is rapidly covered by the SSB protein. The 5' is cleaved more rapidly by the RecB nuclease. **(C)** The RecB nuclease domain promotes the recruitment of the RecA protein to the ssDNA, leading to RecA filament formation. Then, the RecA-ssDNA performs the homology search.

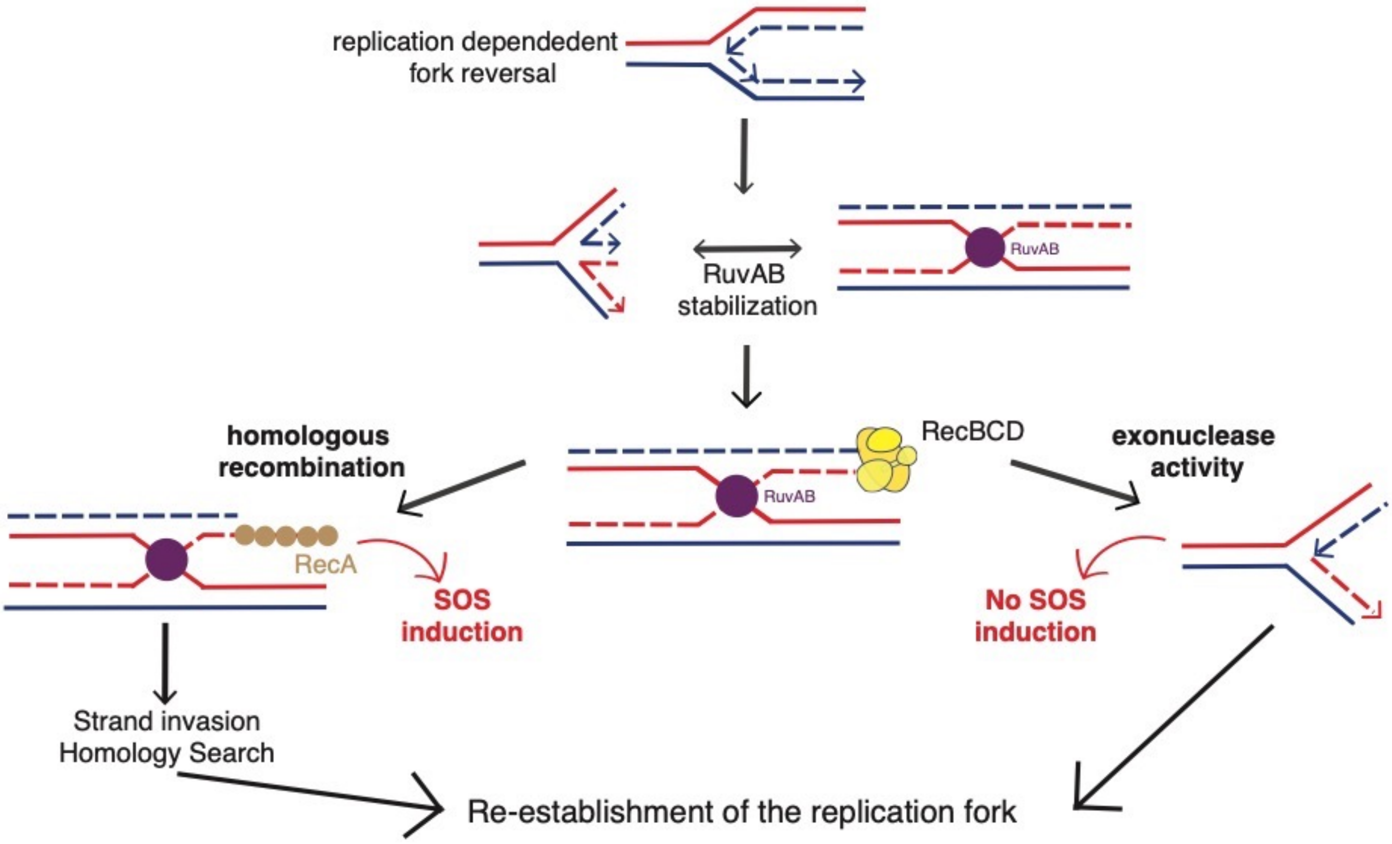

Supplementary Figure 2: Replication fork reversal repair pathways. When replication forks stall, the newly synthesized strands can re-anneal, forming a reversed fork structure. This structure resembles a Holliday junction stabilized by the RuvAB complex. RecBCD acts on the double-stranded DNA ends to restore replication fork progression via two pathways: *exonuclease degradation* (right panel): RecBCD degrades DNA using its exonuclease activity without inducing the SOS response; *homologous recombination* (left panel): RecBCD promotes RecA-loading onto the ssDNA, initiating homologous recombination and leading to SOS induction.

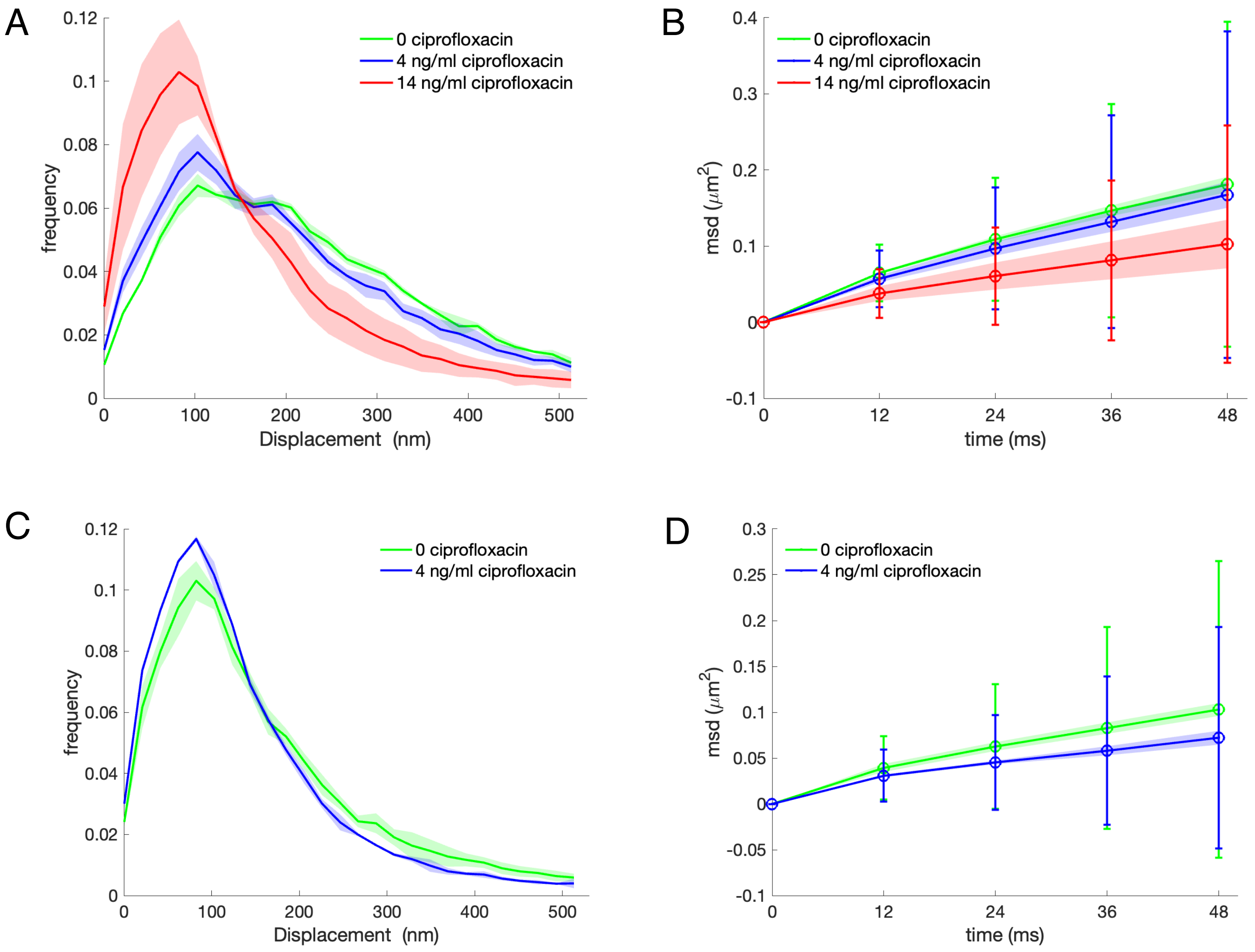

Supplementary Figure 3: Displacement distributions and averaged Mean Square Displacement
(A) WT *recB* averaged displacement distribution**; (B)** WT *recB* averaged MSD with standar deviation bars; **(C)** *recB1080* averaged displacement distribution; **(D)** *recB1080* averaged MSD with standar deviation bars. Averaged datasets: WT *recB* cipro 0; cipro 4 and 14 ng/ml and *recB1080* cipro 0; cipro 4 ng/ml all datasets in Supplementary Table 2. Full lines represent the datasets average, shadow areas the standard deviation.

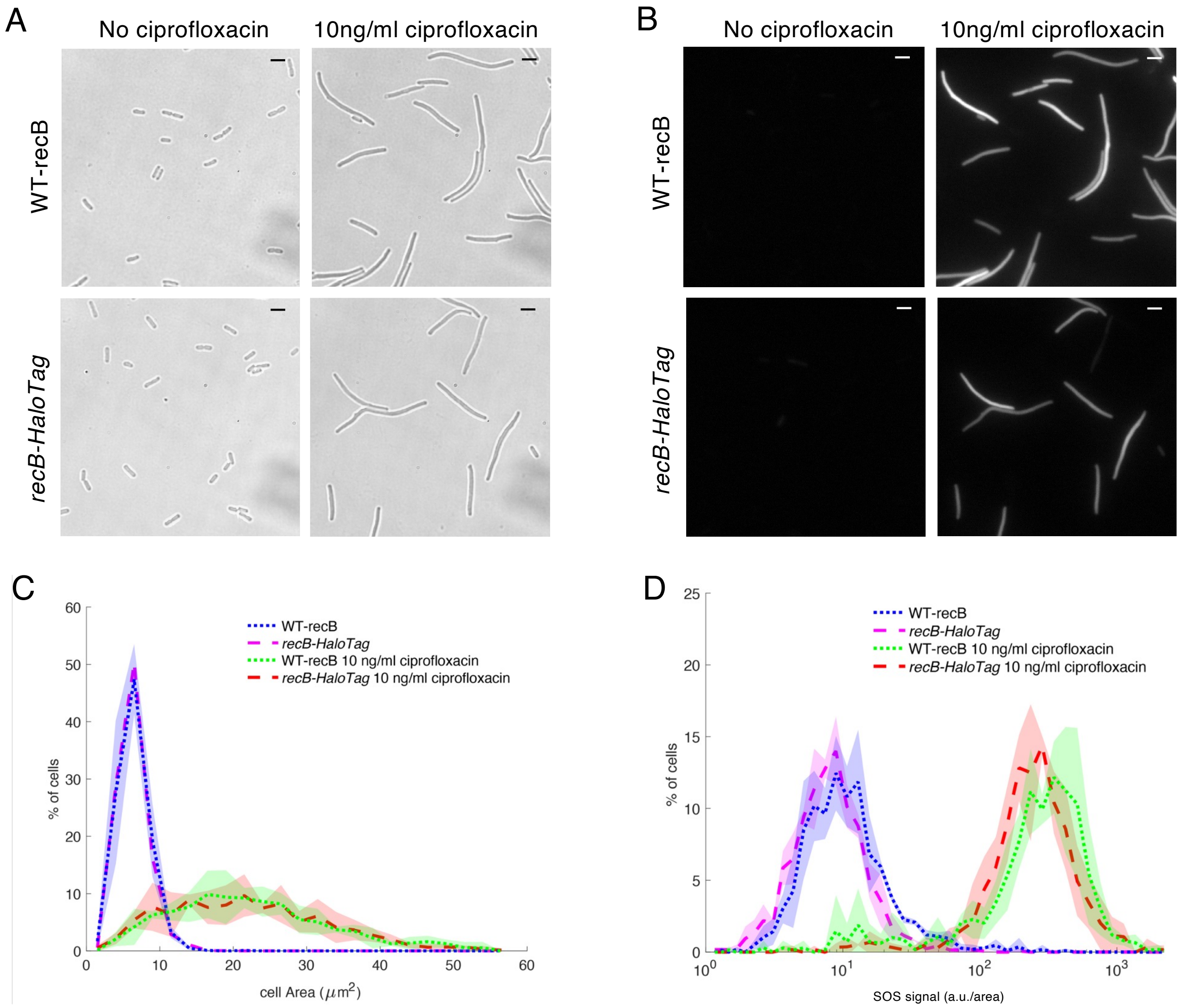

Supplementary Figure 4: HaloTag fusion to RecB does not perturtb SOS response in bacteria cells (A) Representative bright-field and **(B)** SOS induction images of single *E.coli* cells not treated with ciprofloxacin (left top and bottom panels in A and B) and exposed to 10 ng/ml of ciprofloxacin for 150 mins (right top and bottom panels in A and B); top panels: WT-recB strain with SOS reporter *PsulA-mGFP* (MEK455); bottom panels: strain with *recB-HaloTag* fusion and SOS reporter *PsulA-mGFP*(MEK707). Scale bar: 5 µm. **(C)** Bacterial cells area distributions are the same for WT-recB and *recB-HaloTag* strains in both conditions: sample not exposed to ciprofloxacin and sample treated with 10 ng/ml ciprofloxacin. **(D)** SOS signal distributions (GFP per area) is the same in both strain for both conditions: sample not exposed to ciprofloxacin and sample treated with 10 ng/ml ciprofloxacin; averages are computed among three technical replica, dotted lines represent the mean of the three dataset, shadow areas represent the standard deviation.

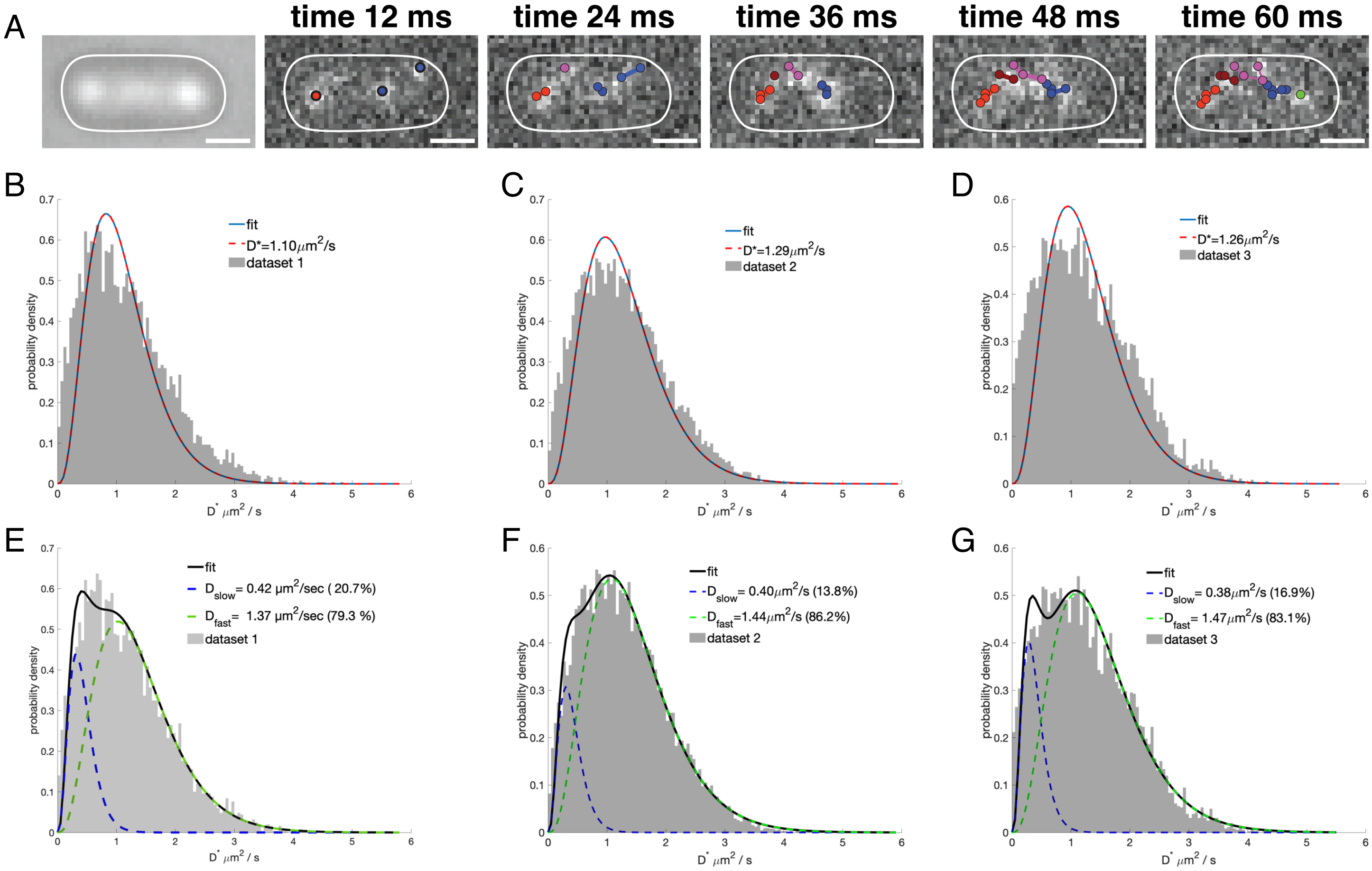

Supplementary Figure 5: (A) Illustration of RecB single-molecule trajectories detected in a single bacterial cell over 5 consecutive frames. Left panel: bright-field. Right panels: progression of the track building overlaid on raw images showing diffraction-limited spots in frames acquired at the indicated time (top of each panel). Same data shown in Figure 1A, here, the raw frames are displayed. Scale bar 1 µm. **Single datasets of RecB-haloTag D* distribution. (B), (C), (D)** D* histograms of RecB-HaloTag datasets with overlayed fit (red curve) of analytical expression of D* described by one population of RecB molecules. **(E), (F)** and **(G)** D* histograms of same datasets shown in panel **(B), (C)** and **(D)** with overlayed fit (full black curve) of D* described as the sum of two sub-populations of RecB molecules with a D^*_{slow} (blues dotted line) and D^*_{fast} (green dotted line). Number of cells and tracks for each dataset in Supplementary Table 2.

Supplementary Note 1: HaloTag imaging

The HaloTag was expressed from the pSF1 plasmid (A. Lepore et al, 2019) containing an arabinose-inducible promoter using 0.01% arabinose. Data acquisition and analysis were performed as described in the Materials and Methods section of the manuscript, except that, due to its high mobility, the exposure time to acquire the streaming video was 8 ms. Given the HaloTag molecule's high mobility, acquiring well-focused videos was challenging, and we had to manually select the videos where we could detect focused diffraction-limited spots.

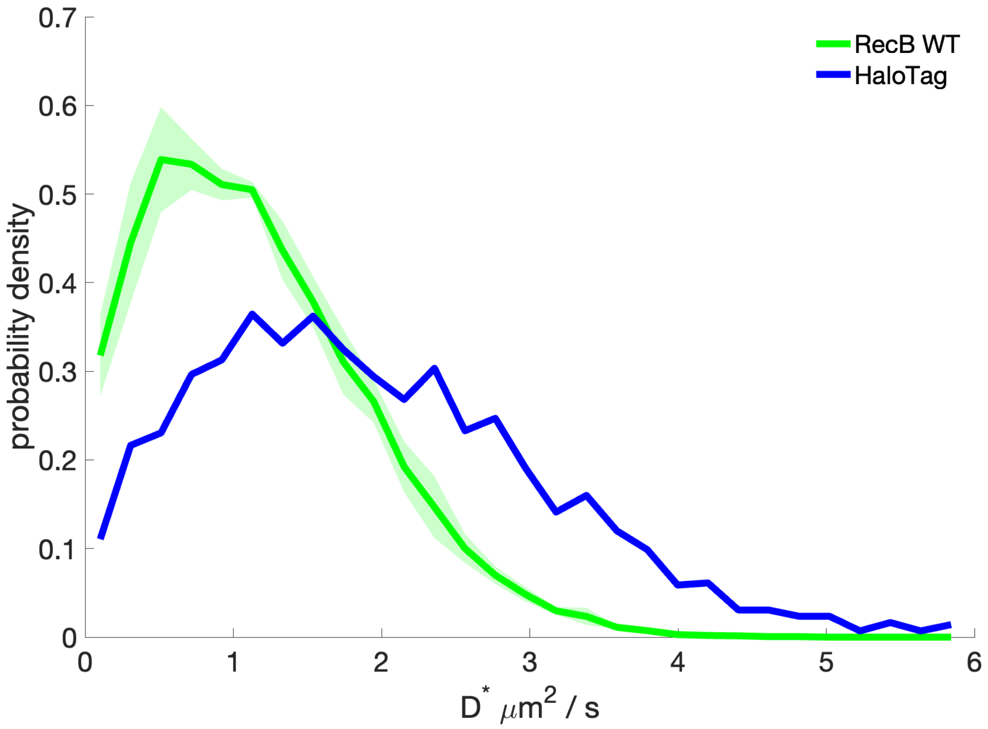

Supplementary Figure 6: The mobility of a DNA-non-interacting protein differs from RecB mobility. Apparent Diffusion coefficient distribution, D*, for the RecB WT strain (same datasets shown in the manuscript Figures 1C and 2B) and for the HaloTag protein ($N_{bacteria}$=101; N_{tracks}=2090). For the RecB WT the full line represent the datasets' average, shadow area the standard deviation.

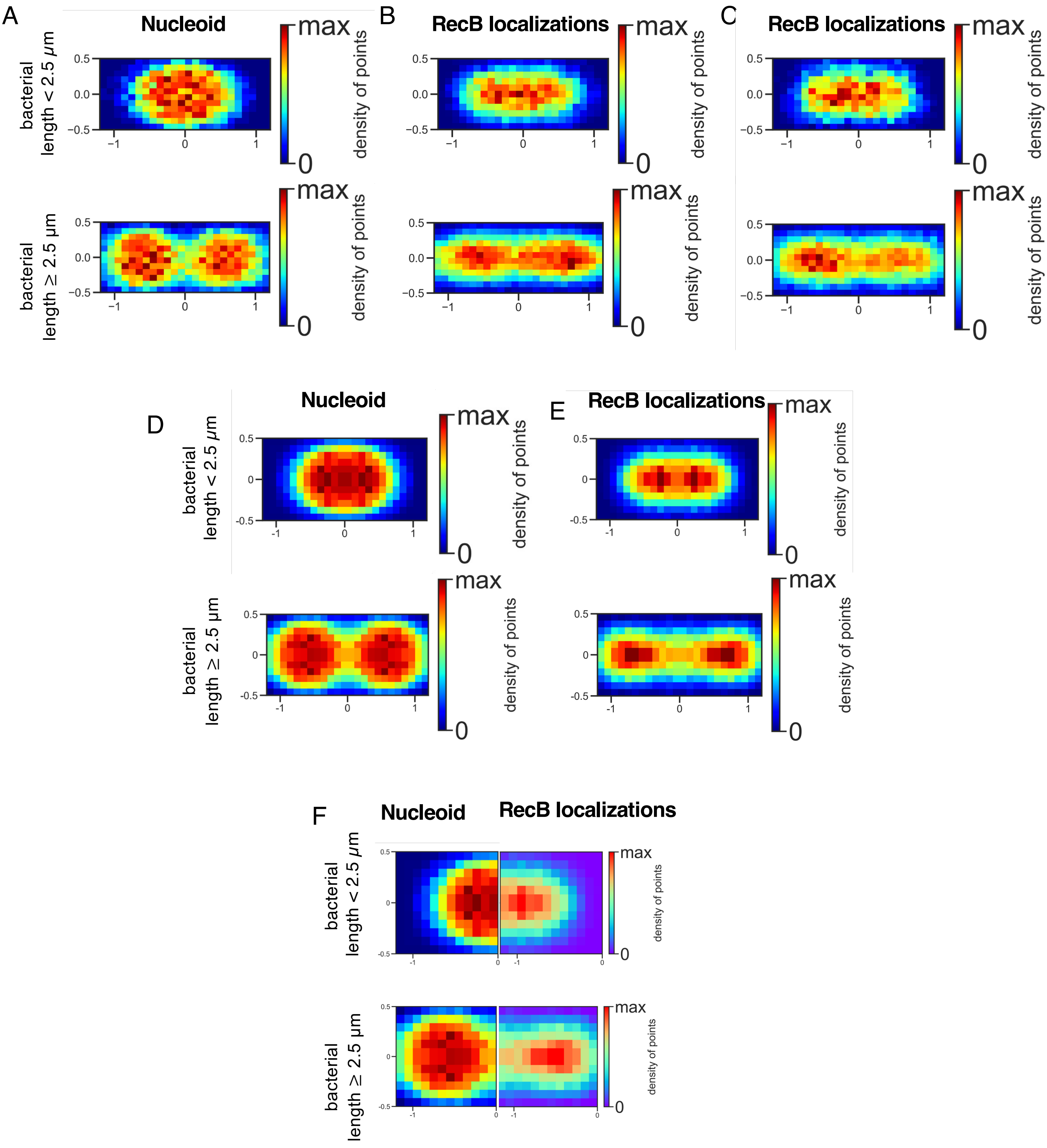

Supplementary Figure 7: RecB colocalize with the nucleoid (A) Nucleoid localization distribution of bacterial DNA stained with SYTOX Green (Number of bacterial cells: 80) normalized by bacterial length and width; **(B)** and **(C)** Localization maps of RecB molecules in bacterial cells of datasets 2 and 3 (same samples as Supplementary Figure 5(C), (D), (F) and (G), see Supplementary Table 2) normalized by bacterial length and width; **Quarter averaged 2D maps** of **(D)** nucleoid localizations, same dataset shown in Figure 1D (right panel) and **(E)** RecB localizations**,** same dataset shown in Figure 1D (left panel); **(F)** comparison of the Nucleoid and RecB localization, same data shown in panels D and E. Top panels (A), (B), (C), (D), (E), (F) : bacterial cells lengths smaller than 2.5 μm ; bottom panels (A), (B), (C), (D), (E), (F) cells lengths equal or longer than 2.5 μm.

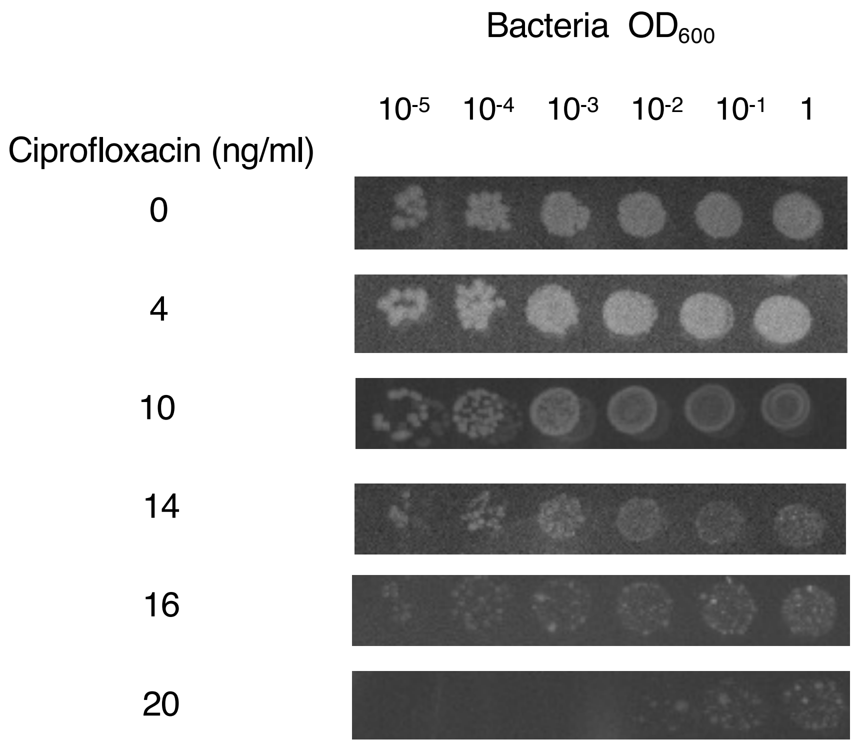

Supplementary Figure 8: Ciprofloxacin sensitivity test of *recB-HaloTag + PsulA-mGFP* (MEK707) strain for different concentrations of ciprofloxacin (see also Supplementary Figure 17).

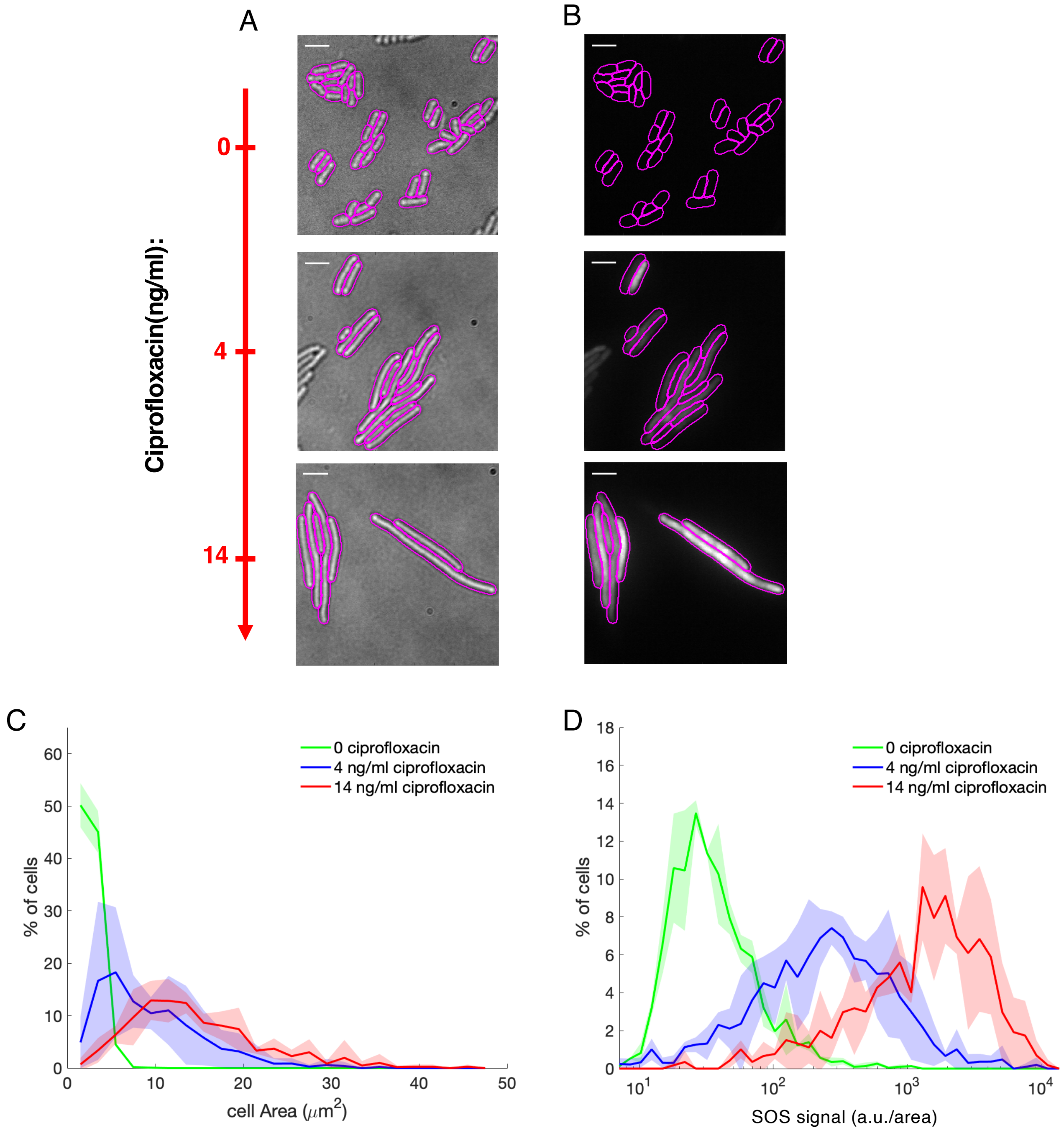

Supplementary Figure 9: **Different sub-lethal concentrations of ciprofloxacin induce distinct levels of DNA damage. (A)** Representative bright-field and **(B)** SOS response induction images of single *recB-HaloTag E.coli* cells with SOS reporter *PsulA-mGFP* (MEK707) for increasing concentrations of ciprofloxacin (from top to bottom). Scale bar: 5 μm. **(C)** cell area distributions and **(D)** SOS induction distributions (GFP per area). Averaged datasets: cipro 0: dataset 2 and dataset 3 (dataset 1 has been excluded for an issue in the GFP channel acquisition), cipro 4 and 14 ng/ml: all datasets in Supp. Table 2. Full lines represent the datasets' average, shadow areas the standard deviation.

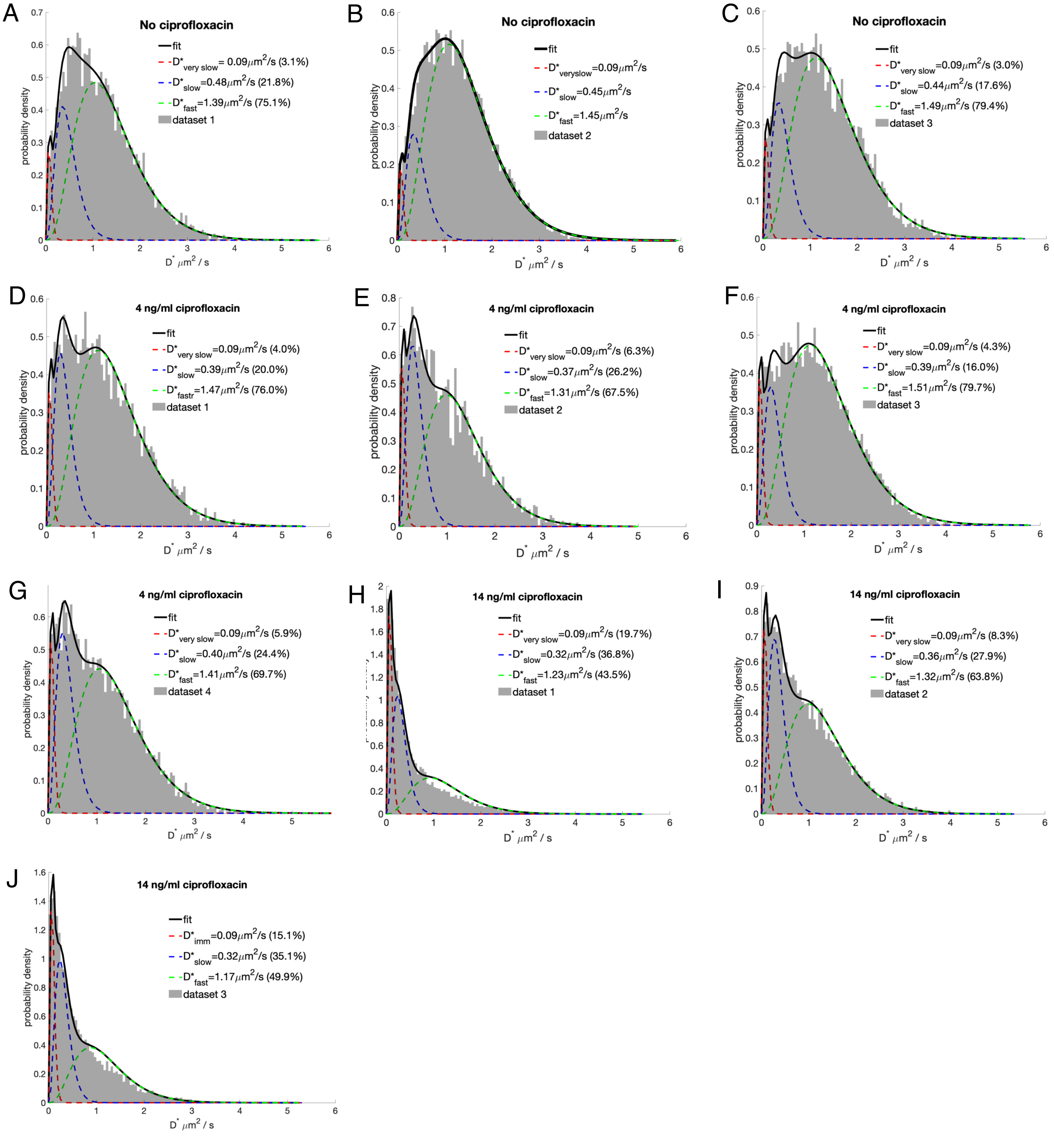

Supplementary Figure 10: Single datasets of RecB-HaloTag D* distributions at 0, 4, 14 ng/ml ciprofloxacin. (A), (B), (C) no ciprofloxacin **(D), (E), (G)** and **(F)** 4 ng/ml of ciprofloxacin;**(H), (I), (J)** 14 ng/ml of ciprofloxacin. See also Supplementary Tables 2 and 4.

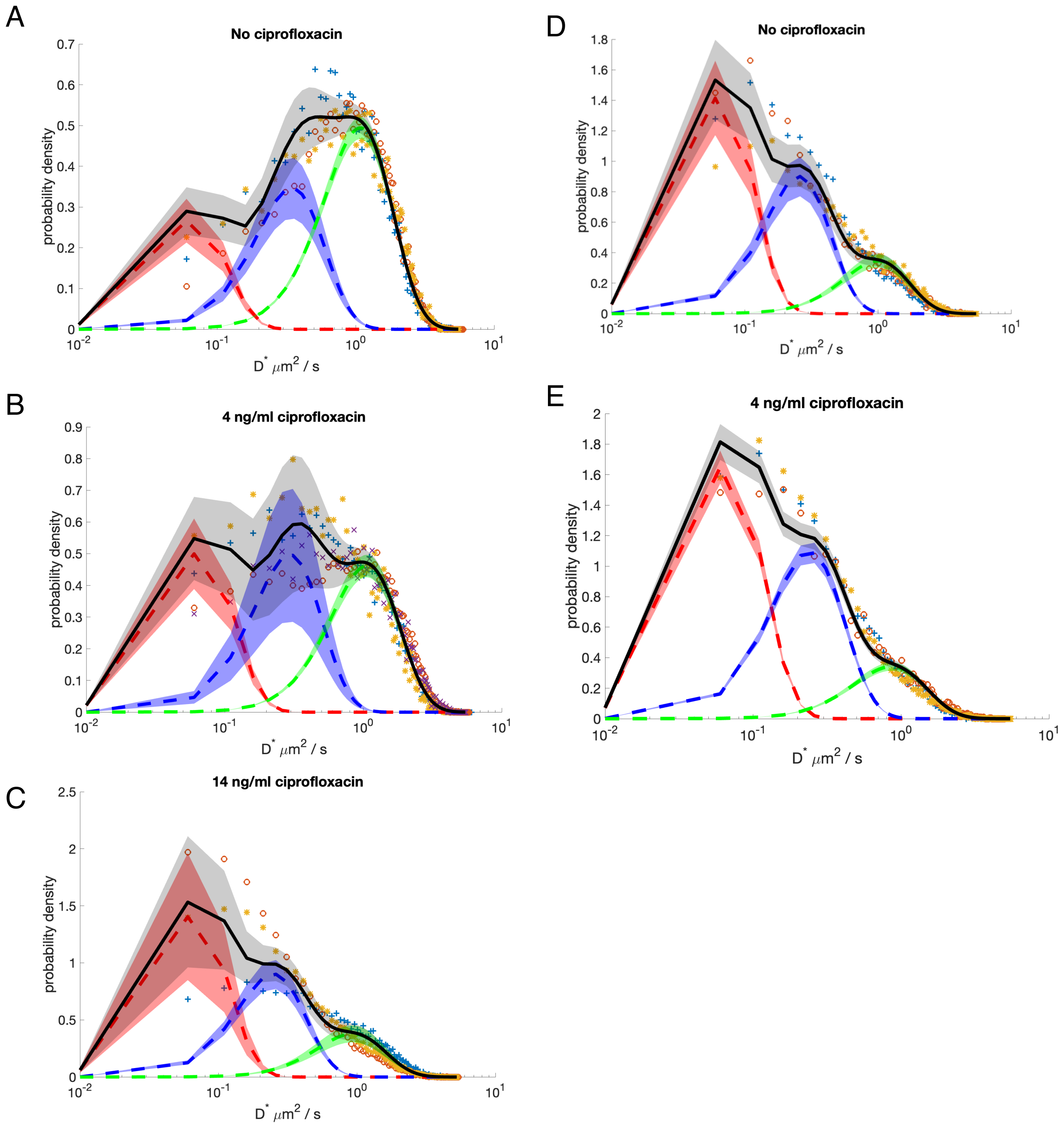

Supplementary Figure 11: D* distributions rapresented in logaritmic scale. (A), **(B)**, **(C)** RecB-HaloTag D* distributions, same datasets shown in Figures 1A, 2B, 2C, 2D; **(D), (E)** RecB1080-HaloTag D* distributions same datasets shown in Figures 4A,4B.

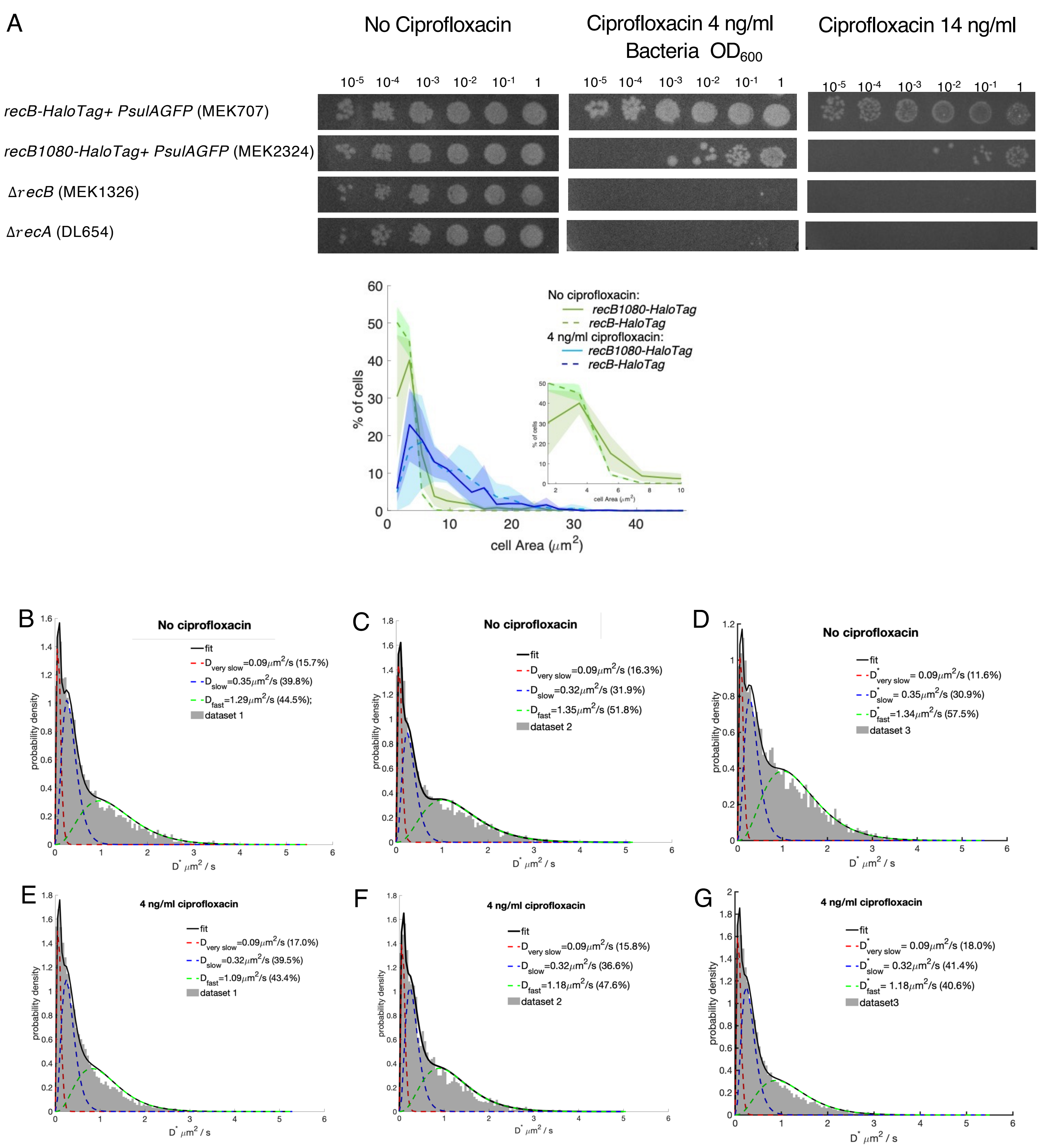

Supplementary Figure 12: ***recB1080-HaloTag* mutant.** (A) Ciprofloxacin sensitivity tests (top panel) and bacterial cell area compared to the *recB-HaloTag* strain (bottom panel) at 0, 4, 14 ng/ml ciprofloxacin. Single datasets D* distributions : **(B), (C)**, **(D)** no ciprofloxacin **(E), (F)** and **(G)** 4 ng/ml of ciprofloxacin. (See also Supplementary Figure 17 and Supplementary Tables 5 and 6).

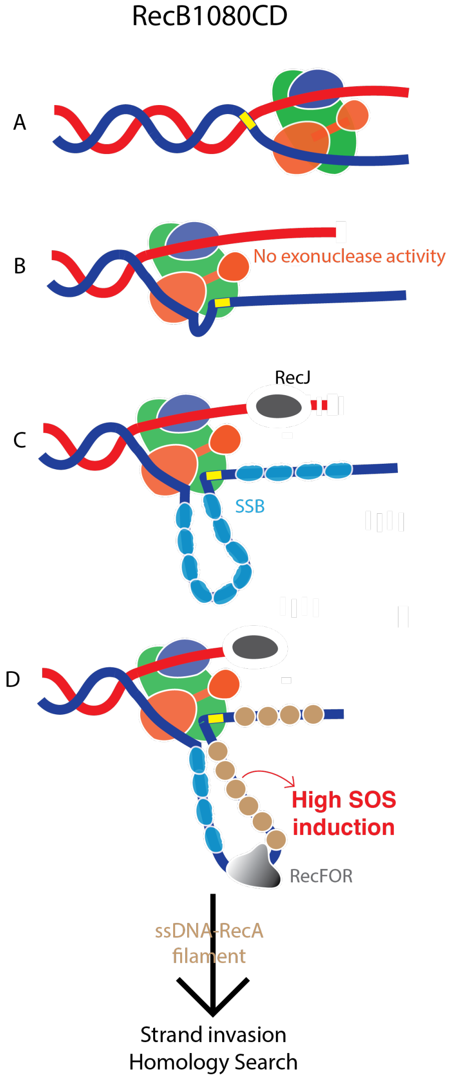

Supplementary Figure 13: **Schematics of the RecB1080CD repair pathway**, based on the models presented in (Churchill et al., 2000; Ivančić-Baće et al. 2003; Singleton et al., 2004). (**A)** RecB1080CD translocates on the dsDNA but does not degrade it; (**B)** RecB1080CD recognizes the $\chi-$ site but does not promote RecA loading. It pauses and possibly undergoes translocation at a reduced rate. Similarly to RecBCD, a loop starts to form upstream of the $\chi-$ site. (**C)** The 3' ssDNA is covered by SSB. The 5' end is degraded by RecJ. Other exonucleases, such as ExoVI (Ivanković et al., 2012), could partially degrade the 3' ssDNA. (**D)** Since RecB1080 lacks nuclease activity, it cannot promote RecA loading, and its continued translocation on the ssDNA could result in a longer 3' end ssDNA. RecA loading is facilitated by RecFOR. After the RecA-ssDNA filament is formed, it performs strand invasion and homology search.

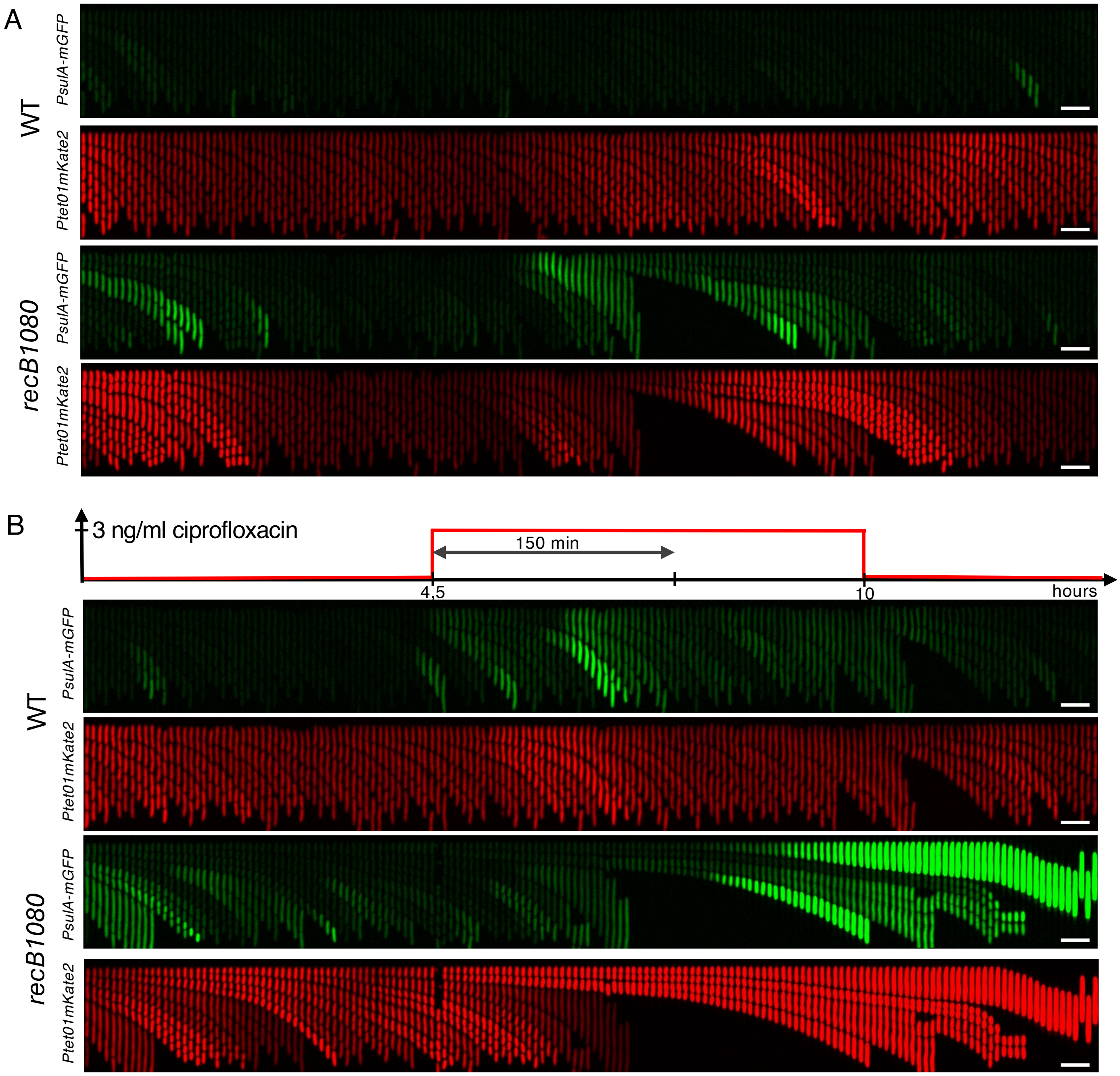

Supplementary Figure 14: SOS signal and constitutive fluorescent reporter expression in the Mother Machine microfluidics (A) Representative kymographs of SOS response induction (*PsulA-mGFP,* in green) and of the constitutive reporter (*Pteto1-mKate2,* in read) of WT and *recB1080* strains not exposed to the antibiotics **(B)** top panel: Schematic showing when the antibiotic was introduced and when the data shown in Figure 3C and D were acquired; bottom panels: representative kymographs of SOS response induction (*PsulA-mGFP,* in green) and of the constitutive reporter (*Pteto1-mKate2,* in read) of WT and *recB1080* strains during following the experiment schematics shown in the panel above. Kymographs are built as a montage of frames with 5-minute intervals from a single microchannel. Scale bar represents 5 µm.

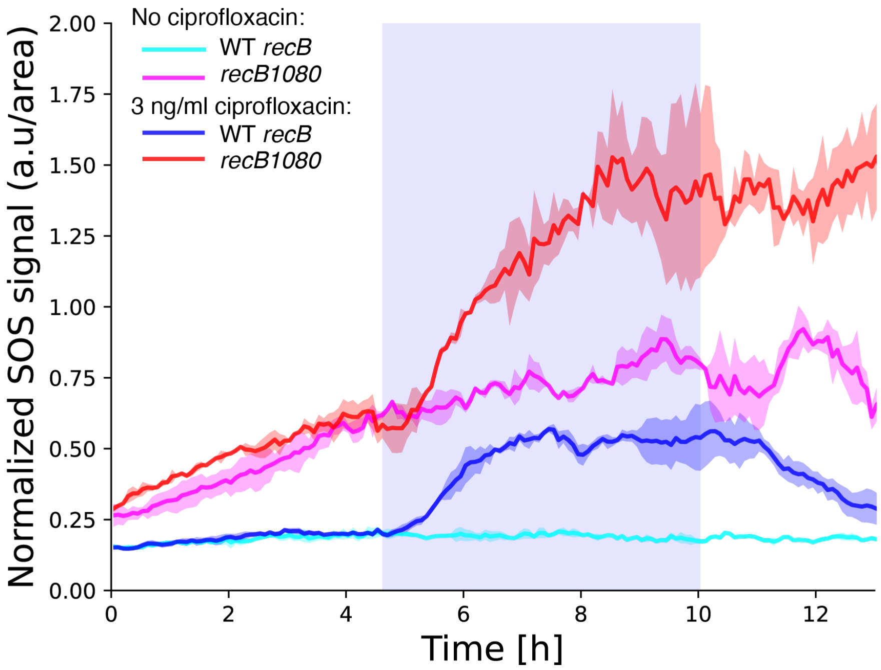

Supplementary Figure 15: Temporal dynamics of the SOS induction in WT and *recB1080.* The plot shows medians of single bacterial cells' normalized SOS signal per cell area over time. To account for possible differences in protein expression over time in each strain and compare the SOS time dynamics in the different strains, we normalized the SOS signal by dividing it by the corresponding signal from the constitutive reporter *Ptet01-mKate2*. In absence of ciprofloxacin, WT SOS signal (cyan) remains constant as expected. *recB1080* strain exhibits constitutively higher SOS levels than WT and increases for the first 4-5 hours, possibly due to repair of endogenous DSBs resulting from replication fork reversal. Ciprofloxacin treatment (3 ng/ml) was initiated at hour 4.5 and removed at hour 10 (blue area). In response to ciprofloxacin, the WT strain (blue) reached a constant SOS expression after approximately 2 hours of exposure and resumed pre-antibiotic levels upon removal. Conversely, recB1080's SOS signal (magenta) increased further with antibiotic exposure and failed to recover after ciprofloxacin removal. Shadow areas around full lines represent the standard deviation from the median.

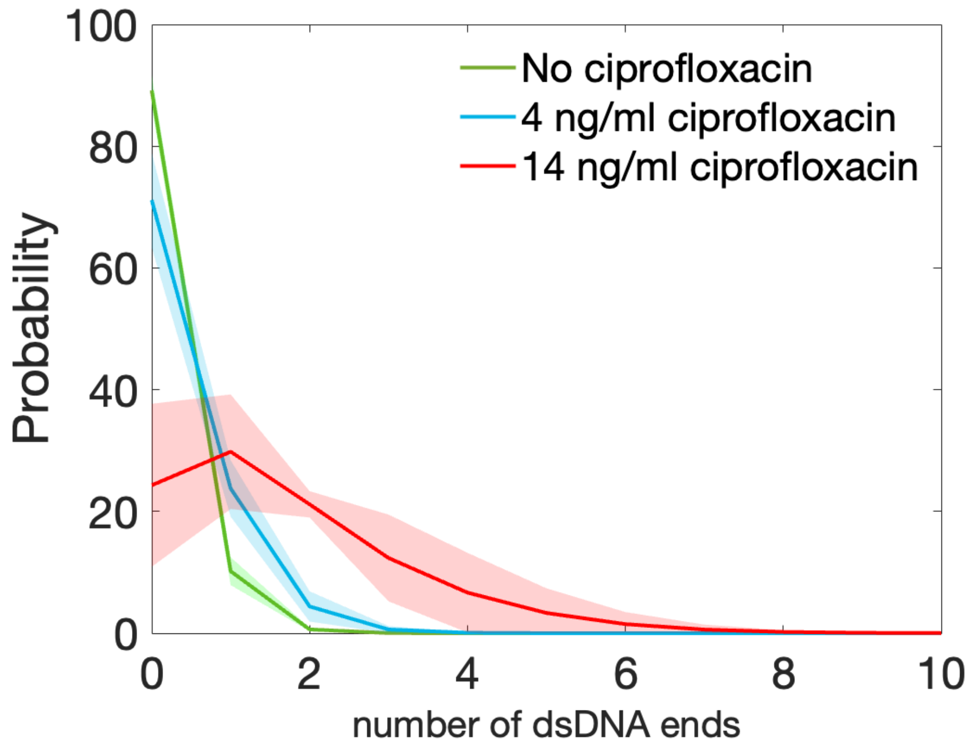

Supplementary Figure 16: Poisson distribution describing the probability of having bacterial cell with one, two or more dsDNA ends in the *recB* WT strain for no antibiotic and at sub-lethal concentrations of ciprofloxacin. The percentage of bacterial cells with no RecB on the dsDNA ends was estimated using the percentages of bacterial cells with at least one RecB on the DNA substrate for each datates, then averaged (as done to made Table 2). The distribution parameter was then computed based on the probability of RecB not being present on the DNA. Solid lines represent the averages between dataset and the shadow area the standard deviation from the average.

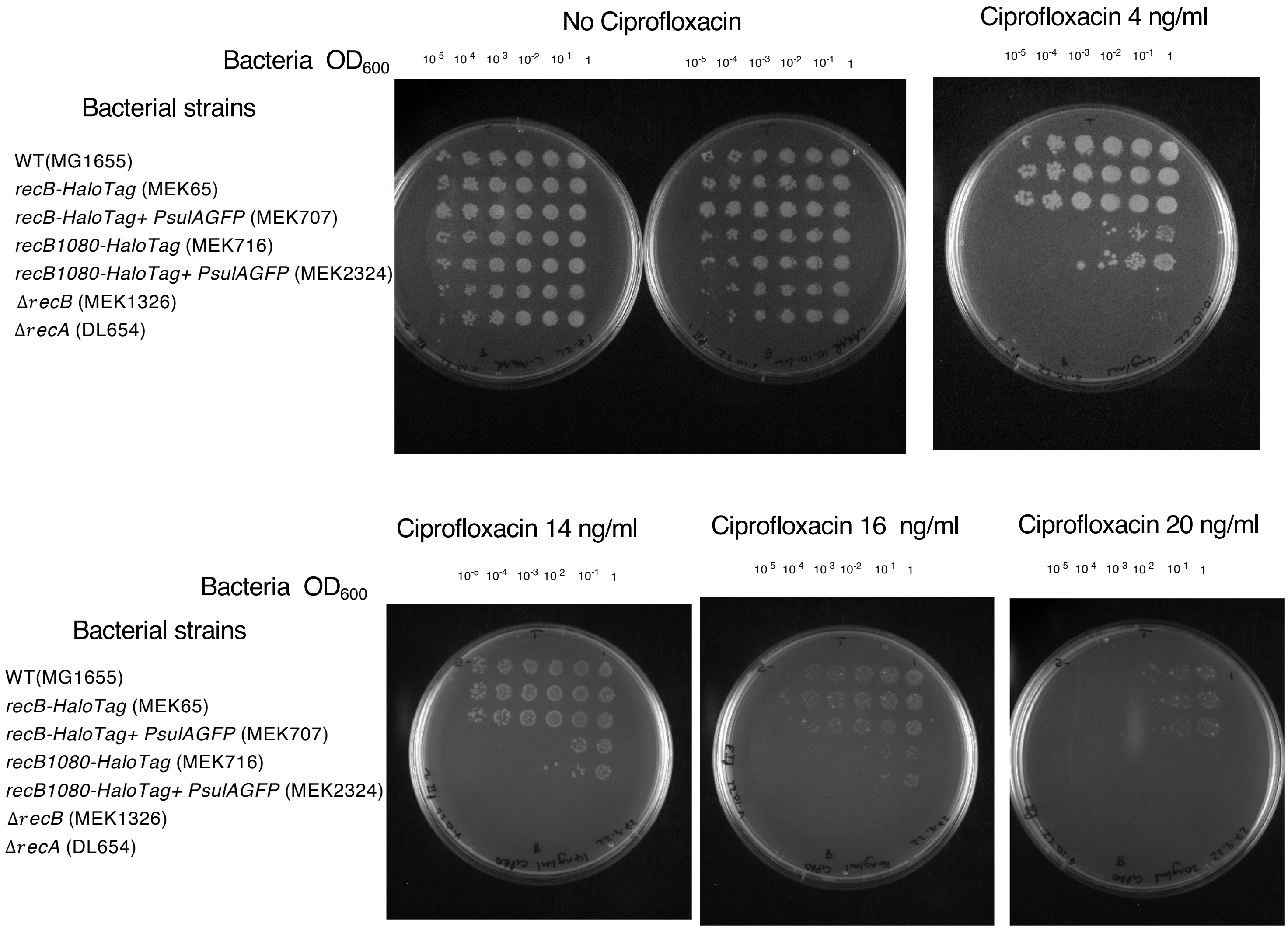

Supplementary Figure 17: Ciprofloxacin sensitivity tests (original plates images).

Primer	5'- 3' sequence	Purpose
oSJR058	GGAATCAATGCCTGAGTG	pOSIP HK022 insertion verification
oSJR061	GGCATCAACAGCACATTC	pOSIP HK022 insertion verification
oSJR116	AGGGTTGATCTTTGTTGT	PsfiA insertion verfication
oSJR021	ATTTAAGAAGGAGATATACAT	mGFP insertion verification
Rec1080G-1	AAAAACTGCAGAACCGACGTTAACACCACATC	pDL4174 construction
Rec1080G-2	GGATTTATAGGCCAGCAGGTA	pDL4174 construction
Rec1080G-3	TACCTGCTGGCCTATAAATCC	pDL4174 construction
Rec1080G-4	AAAAAGTCGACTGTTTGTGCTCCACAGCTTC	pDL4174 construction
pKOF	AGGGCAGGGTCGTTAAATAGC	*recB1080* insertion verification
pKOR2	AGGGAAGAAAGCGAAAGGAG	*recB1080* insertion verification

Supplementary Table 1 : Oligos used in strain construction

ciprofloxacin concentration(ng/ml)	Number of bacteria	Number of tracks
0 - dataset 1	1127	5001
0 - dataset 2	1118	12937
0 - dataset 3	585	7196
4 - dataset 1	288	5328
4 - dataset 2	128	4036
4 - dataset 3	471	11379
4 - dataset 4	305	6465
14 - dataset 1	182	14874
14 - dataset 2	136	10115
14 - dataset 3	192	11204

Supplementary Table 2 : *recB-HaloTag* datasets. Number of bacteria and detected tracks for each dataset

	D* (µm^2/sec)		% of D* trajectories	
ciprofloxacin concentration(ng/ml)	slow	fast	slow	fast
0 - dataset 1	0.42	1.37	20.7	79.3
0 - dataset 2	0.40	1.44	13.8	86.2
0 - dataset 3	0.38	1.47	16.9	83.1
0 - average +/- std	0.40 +/- 0.02	1.43+/- 0.05	17.1 +/- 3.4	82.9 +/- 3.4

Supplementary Table 3 : two sub-population fit results of RecB-HaloTag D* distributions for all the datasets without ciprofloxacin

	D* (µm^2/sec)			% of D* trajectories		
ciprofloxacin concentration(ng/ml)	very slow	slow	fast	very slow	slow	fast
0 - dataset 1	0.09	0.48	1.39	3.1	21.8	75.1
0 - dataset 2	0.09	0.45	1.45	2.1	14.1	83.8
0 - dataset 3	0.09	0.44	1.49	3.0	17.6	79.4
0 - average +/- std	0.09	0.46 +/- 0.02	1.44 +/- 0.05	2.7 +/- 0.6	17.8+/- 3.9	79.4 +/- 4.4
4 - dataset 1	0.09	0.40	1.41	5.9	24.4	69.7
4 - dataset 2	0.09	0.39	1.51	4.3	16	79.7
4 - dataset 3	0.09	0.37	1.31	6.3	26.2	67.5
4 - dataset 4	0.09	0.39	1.47	4.0	20	76
4 - average +/- std	0.09	0.39 +/- 0.01	1.42 +/-0.09	5.1 +/- 1.1	21.7 +/- 9.2	73.2 +/- 5.6
14 - dataset 1	0.09	0.32	1.17	15.1	35.1	49.9
14 - dataset 2	0.09	0.36	1.32	8.3	27.9	63.8
14 - dataset 3	0.09	0.32	1.23	19.7	36.8	43.5
14 - average +/- std	0.09	0.33 +/- 0.02	1.24 +/- 0.07	14.4 +/- 5.7	33.3 +/- 4.7	52.4 +/-10.3

Supplementary Table 4 : three sub-population fit results of RecB-HaloTag D* distributions for all the datasets

ciprofloxacin concentration(ng/ml)	Number of bactiera	Number of tracks
0 - dataset 1	206	5086
0 - dataset 2	326	3721
0 - dataset 3	700	7637
4 - dataset 1	266	12328
4 - dataset 2	329	4158
4 - dataset 3	537	10375

Supplementary Table 5 : *recB1080-HaloTag* datasets. Number of bacteria and detected tracks for each dataset

	D* (μm^2/sec)			% of D* trajectories		
ciprofloxacin concentration(ng/ml)	very slow	slow	fast	very slow	slow	fast
0 - dataset 1	0.09	0.35	1.29	15.7	39.8	44.5
0 - dataset 2	0.09	0.32	1.35	16.3	31.9	51.8
0 - dataset 3	0.09	0.35	1.34	11.6	30.9	57.5
0 - average +/- std	0.09	0.34 +/- 0.2	1.33 +/-0.03	14.5+/- 2.5	34.2+/- 4.9	51.3+/-6.5
4 – dataset 1	0.09	0.32	1.09	17	39.5	43.4
4 - dataset 2	0.09	0.32	1.18	15.8	36.6	47.6
4 - dataset 3	0.09	0.32	1.18	18	41.4	40.6
4 - average +/- std	0.09	0.32 +/- 0	1.15 +/-0.05	16.9 +/- 1.1	39.2 +/- 2.4	43.9+/-3.5

Supplementary Table 6 : three sub-population fit results of RecB1080-HaloTag D* distributions for all the datasets
